# Genetic Dissection of Morphometric Traits Reveals That Phytochrome B Affects Nucleus Size and Heterochromatin Organization in *Arabidopsis thaliana*

**DOI:** 10.1534/g3.117.043539

**Published:** 2017-06-06

**Authors:** Basten L. Snoek, Penka Pavlova, Federico Tessadori, Anton J. M. Peeters, Clara Bourbousse, Fredy Barneche, Hans de Jong, Paul F. Fransz, Martijn van Zanten

**Affiliations:** * Laboratory of Nematology, Wageningen University, 6708 PB, The Netherlands; ‡ Laboratory of Genetics, Wageningen University, 6708 PB, The Netherlands; † Theoretical Biology and Bioinformatics, Institute of Biodynamics and Biocomplexity; †† Department of Biology, Institute of Education, Utrecht University, 3584 CH, The Netherlands; §§ Molecular Plant Physiology, Institute of Environmental Biology, Utrecht University, 3584 CH, The Netherlands; § Plant Development and (Epi)Genetics, Swammerdam Institute for Life Sciences, University of Amsterdam, 1098 SM, The Netherlands; ** Hubrecht Institute, Royal Netherlands Academy of Arts and Sciences, University Medical Center Utrecht, 3584 CT, The Netherlands; ‡‡ Institut de Biologie de l’Ecole Normale Supérieure, Centre National de la Recherche Scientifique, Unité Mixte de Recherche 8197, Institut National de la Santé et de la Recherche Médicale U1024, Ecole Normale Supérieure, Paris Sciences et Lettres Research University, F-75005, France

**Keywords:** chromocenter, Phytochrome B, nucleus size, QTL analysis, *Arabidopsis thaliana*

## Abstract

Microscopically visible chromatin is partitioned into two major components in *Arabidopsis thaliana* nuclei. On one hand, chromocenters are conspicuous foci of highly condensed “heterochromatic” domains that contain mostly repeated sequences. On the other hand, less condensed and gene-rich “euchromatin” emanates from these chromocenters. This differentiation, together with the dynamic nature of chromatin compaction in response to developmental and environmental stimuli, makes *Arabidopsis* a powerful system for studying chromatin organization and dynamics. Heterochromatin dynamics can be monitored by measuring the Heterochromatin Index, *i.e.*, the proportion of nuclei displaying well-defined chromocenters, or the DNA fraction of chromocenters (relative heterochromatin fraction). Both measures are composite traits, thus their values represent the sum of effects of various underlying morphometric properties. We exploited genetic variation between natural occurring accessions to determine the genetic basis of individual nucleus and chromocenter morphometric parameters (area, perimeter, density, roundness, and heterogeneity) that together determine chromatin compaction. Our novel reductionist genetic approach revealed quantitative trait loci (QTL) for all measured traits. Genomic colocalization among QTL was limited, which suggests a complex genetic regulation of chromatin compaction. Yet genomic intervals of QTL for nucleus size (area and perimeter) both overlap with a known QTL for heterochromatin compaction that is explained by natural polymorphism in the red/far-red light and temperature receptor Phytochrome B. Mutant analyses and genetic complementation assays show that Phytochrome B is a negative regulator of nucleus size, revealing that perception of climatic conditions by a Phytochrome-mediated hub is a major determinant for coordinating nucleus size and heterochromatin compaction.

The eukaryotic chromosome is composed of a long chromatin fiber consisting of DNA bound by histones and many different nonhistone proteins that control chromosome organization and gene activity. Several levels of chromatin folding are required to fit the DNA in the confined space of the nucleus and to allow differential access of the transcription machinery and transcription regulatory factors to the DNA (Fransz and de Jong 2011). Based on classical microscopic staining methods, two types of chromatin states, euchromatin and heterochromatin, were distinguished (Heitz 1928, 1929). DNA fluorescence *in situ* hybridization (FISH), protein immunolabeling, and epigenomic methodologies have revealed that euchromatin and heterochromatin are genetically and biochemically distinct. While euchromatin is gene-rich and heavily marked by acetylated histones and histone H3 lysine 4 methylation (H3K4me), the brightly stained heterochromatin is rich in repetitive sequences, heavily marked by H3K9 methylation and 5-methylcytosine, and is largely transcriptionally inactive (Fransz *et al.* 2006; Fransz and de Jong 2011).

Despite improved chromatin staining methods, reliable quantification of condensation states remains difficult as in many species the boundaries between visible heterochromatin and euchromatin are blurred (Brown 1966). The model plant species *Arabidopsis thaliana* is one exception, with a characteristic and discernible chromatin organization at the light microscopic level. In most *Arabidopsis* cell nuclei, including those of leaf mesophyll cells, typically 8–10 discrete and brightly stainable chromocenters can be recognized, in which most heterochromatin aggregates (Fransz *et al.* 2002; Tessadori *et al.* 2004). DNA-FISH experiments demonstrated that chromocenters in *Arabidopsis* are formed around the 180-bp centromeric repeats and pericentromeric domains and contain the major repeat fraction of the genome, including silent 45S rDNA tandem repeats from the nucleolar organizing region (NOR) of chromosome 2 and 4 (Fransz *et al.* 2002; Chandrasekhara *et al.* 2016). Gene-rich euchromatic loops that are prone to transcriptional regulation emanate from these chromocenters. Chromocenters generally spatially localize near to the nuclear periphery (Poulet *et al.* 2017) and may consequently influence the whole genome topology (Liu and Weigel 2015).

Based on 4′,6′-diamidino-2-phenylindole (DAPI) phenotypes of chromatin compaction patterns, three types of nuclei have been recognized in *Arabidopsis* (Tessadori *et al.* 2009; van Zanten *et al.* 2010; Bourbousse *et al.* 2015): type 1 (condensed) nuclei contain 8–10 brightly stained, distinct round chromocenters; type 2 (intermediate) nuclei contain irregular shaped, diffuse chromocenters; and type 3 (decondensed) nuclei lack visual chromocenters. The two NOR-containing chromocenters flanking the nucleolus usually remain compacted under chromocenter-destabilizing contexts. The fraction of type 1 nuclei is used as a Heterochromatin Index (HX) to arbitrarily estimate chromatin compaction levels in a given population of nuclei (Tessadori *et al.* 2009; van Zanten *et al.* 2010; Wang *et al.* 2013; Yelagandula *et al.* 2014). A more quantitative measure of chromatin condensation levels can be obtained by computational image analysis of individual nuclei by defining the relative heterochromatin fraction (RHF). This trait represents the sum of DNA fluorescence intensity (density) of chromocenters relatively to the entire nucleus, and hence, represents the proportion of DNA found within chromocenters (Soppe *et al.* 2002; Tessadori *et al.* 2009; Pavlova *et al.* 2010; van Zanten *et al.* 2010).

The use of the composite traits HX and/or the RHF has been the basis of several studies aimed at investigating *Arabidopsis* heterochromatin organization. For instance, analysis of RHF revealed strong aggregation of heterochromatin in the small-sized nuclei of embryonic cotyledons during seed maturation. Upon subsequent steps of seed germination, the nuclear size increases extensively. Heterochromatin is first decompacted during germination (van Zanten *et al.* 2011) and subsequently recompacted into chromocenters when photomorphogenic seedlings establish and cotyledon cells differentiate (Mathieu *et al.* 2003; van Zanten *et al.* 2011; Bourbousse *et al.* 2015). Compaction progresses when leaves mature (Tessadori *et al.* 2004). Accordingly, artificial dedifferentiation of mesophyll cell nuclei during protoplast culturing leads to a severe decompaction of the chromocenters (Tessadori *et al.* 2007a).

Fluctuations in heterochromatin compaction also occur during developmental phase transitions in the mature plant. Prior to bolting, a transient decline in the level of heterochromatin compaction mediated by the photoreceptor cryptochrome 2 is observed in the rosette leaves (Tessadori *et al.* 2007b). In addition, chromatin compaction is strongly influenced by biotic (*e.g.*, pathogens; Pavet *et al.* 2006), and abiotic conditions (*e.g.*, heat; Pecinka *et al.* 2010; Wang *et al.* 2013), and notably, by light (Barneche *et al.* 2014; Probst and Mittelsten Scheid 2015; Perrella and Kaiserli 2016). Light perception by photoreceptors is essential for both nucleus growth and chromocenter formation during seedling cotyledon development, by releasing CONSTITUTIVE PHOTOMORPHOGENIC 1–dependent repression of heterochromatin condensation in darkness (Bourbousse *et al.* 2015). At later developmental stages, chromocenters in leaf nuclei also severely disaggregate when plants are subjected to suboptimal light conditions, such as low light intensities, low blue light, or light with a low red/far-red ratio (Tessadori *et al.* 2009; van Zanten *et al.* 2010, 2012). Conversely, light intensity is limiting for chromocenter formation in the subtropical *Arabidopsis* accession Cvi-0, as compared to laboratory strains Col-0 and L*er* (Tessadori *et al.* 2009).

Despite extensive use of HX and RHF, full molecular understanding of chromocenter formation and (de)condensation events is hampered by the fact that these measures are the result of an interplay between several morphometric parameters, such as sizes and shapes of both nucleus and chromocenters. Here, we investigated the genetic basis of nuclear organization in *A. thaliana* in a novel reductionist manner, by using refined quantitative morphometric analyses of area, perimeter, density, heterogeneity, and roundness. We examined the genetic architecture of these five morphometric traits in recombinant inbred lines (RILs) of the L*er* × Cvi-0 population (Alonso-Blanco *et al.* 1998). This revealed quantitative trait loci (QTL) for all individual morphometric traits with little overlap among genomic QTL positions, indicating that chromatin organization has a highly complex genetic basis. We conclude that multiple loci are involved in the genetic regulation of morphometry traits of nucleus and chromocenters that together contribute to chromatin (de)condensation. Confirmed morphometric QTL for nucleus area and perimeter colocated with the previously mapped negative-effect RHF QTL on chromosome 2 (RHF2) (Tessadori *et al.* 2009). In this previous work, we demonstrated that RHF2 could be explained by natural genetic variation in the photo- and thermoreceptor Phytochrome B (PhyB) (Koornneef *et al.* 1980; Jung *et al.* 2016; Legris *et al.* 2016). Here we present mutant and genetic data indicating that PhyB is a negative regulator of nucleus size (area and perimeter) independent of its role in chromocenter formation and/or maintenance. Given the strong correlation between RHF on one hand and nucleus area and perimeter on the other, we propose that PhyB activity is a central component in the coregulation of nucleus size and heterochromatin condensation levels.

## Materials and Methods

### Plant materials and growth conditions

Plant materials and growth conditions were as described in (Tessadori *et al.* 2009) unless stated otherwise. Seeds of Cvi-0 (N902), L*er* (NW20), *phyb-5* (N69) (Koornneef *et al.* 1980), and *phyb-9* (N6217) (Reed *et al.* 1993) were obtained from Nottingham *Arabidopsis* Stock Centre. The Cvi-0 × L*er* RILs (Alonso-Blanco *et al.* 1998) and near isogenic lines (NILs) (Keurentjes *et al.* 2007) were kindly provided by M. Koornneef (Wageningen University, Wageningen, The Netherlands).

For morphometry trait measurements, correlations and QTL analyses, 3–4-wk-old RILs of the L*er* × Cvi-0 core population (Alonso-Blanco *et al.* 1998), parental lines, and hybrid Cvi-0 × L*er* F1s, derived from crossing, were selected for fixation of the rosettes (see section Leaf nuclei spread preparations). Plants were grown in a greenhouse in 16 hr light/8 hr darkness on standard potting soil in a randomized design. These long day conditions induce the potential for flowering, a trait known to be segregating in the RIL set used (Alonso-Blanco *et al.* 1998), and was chosen to synchronize this trait, thereby circumventing interference of floral transition–related chromatin reorganization (Tessadori *et al.* 2007b).

NILs for QTL confirmation, Cvi-0, L*er*, *phyb-5*, and hybrid F1, for mutant and genetic complementation analysis were grown in controlled, in-house, growth cabinets (20°, 70% v/v relative humidity during day and night) under long day conditions, with 150–200 μmol m^-2^ s^-1^ photosynthetic active radiation. Plants were harvested 3 wk after germination.

(De)etiolation experiments were performed as previously described (Bourbousse *et al.* 2015). Sterilized seeds were sown on filter papers on top of MS medium supplemented with 0.9% agar. Germination was induced by exposing imbibed seeds to white light for 4 hr and subsequently shifting to darkness. After 4 d, half of the plates were put into white light (100 µmol m^-2^ s^-1^) conditions 24 hr before harvesting, while the other half remained in darkness for the full 5 d.

### Leaf nuclei spread preparations

Rosette leaf material was harvested and directly fixed in ice-cold Carnoy’s fixative (3:1 ethanol/acetic acid) and kept at –20° until use. Nuclei spread preparations for microscopic analysis were made essentially as described by Schubert *et al.* (2001) and Tessadori *et al.* (2009) using modified enzymatic cell wall–degrading mixture [cellulose Onozuka R10 (Yakult), 0.25% macerozyme R10 (Duchefa) in 10 mM citrate buffer, pH 4.5] to digest cell walls. The air-dried slides were mounted in Vectashield (Vector Laboratories) with DAPI (2 µg ml^-1^) before observation and capturing.

For the (de)etiolation experiments, cotyledons of 5-d-old seedlings were fixed in 4% paraformaldehyde for 3 hr under white light condition or under a safe green light for the dark-grown seedlings, and treated with a solution containing 0.5% cellulose Onozuka R10 (Yakult), 0.25% macerozyme R10 (Duchefa), and 0.1% Triton X-100 for 1 hr. Cotyledons from at least three seedlings were isolated and squashed on a glass slide, flash frozen in liquid nitrogen, and incubated with PEMSB (50 mM Pipes, pH 7.3; 5 mM EGTA, pH 7.1; 5 mM MgSO_4_; 0.05% saponin; 5% wt/vol BSA) before being mounted with Vectashield (Vector Laboratories) supplemented with 2 μg/ml DAPI before observation and capturing.

### Quantitative morphometric analysis of chromatin and nuclei

Between 30 and 100 nuclei per sample were captured with an Olympus BX6000 epifluorescence microscope (Olympus, Tokyo, Japan) coupled to a CCD camera (Coolsnap FX; Photometrics, Tucson, AZ). Images were captured underexposed (30–50% of the gray scale) for a high accuracy in measuring the intensity of the pixels. Subsequently, images were exported as 8 bit in TIFF format for quantitative analysis and analyzed using a (semi)automatic macro, developed in-house in Image ProPlus (Media Cybernetics, Bethesda, MD). A detailed description of this macro and macro script are available in Pavlova *et al.* (2010). In short, outlines of the nucleus and the individual chromocenters (“finding edges”) were established by a threshold algorithm and next the following primary morphometric parameters were analyzed for both nuclei and chromocenters: area (area of the object, excluding holes in pixels), density (averaged optical density), perimeter (length of the object’s outline in pixels), roundness (relative deviation from a perfect circle), and heterogeneity (fraction of pixels that deviate >10% from the averaged intensity). All data were exported to Microsoft Excel and further processed and analyzed in this software package. An overview of the primary and derived morphometric parameters used in this study is presented in Table 1.

**Table 1:** Nucleus (*nu*) and chromocenter (*cc*) morphometric parameters described in this study

Trait	Description	Formula	Unit
Area	Area of *nu* or *cc*	Ʃ pixels of *nu* or *cc*	Pixel
Intensity	Average fluorescence intensity of DAPI stain of *nu* or *cc*		
Perimeter	Length of the object’s outline	Ʃ pixels	Pixel
Roundness	Circularity of *nu* of *cc* (value of one means round)	(Perimeter_*nu*^^2^) / (4×pi×Area_*nu*) or (Perimeter_*cc*^^2^) / (4×pi×Area_*cc*)	
Heterogeneity	Fraction of pixels that deviate >10% from the averaged intensity of *nu* or *cc*		%
RHF	Fraction *cc* per *nu*	[Area_*cc*×(Intensity_*cc*_Intensity_background)]/ [Area_*nu*×(Intensity_*nu*_Intensity_background)]	%

For the skoto- and photomorphogenesis (dark + 24 hr light) experiments, *Z*-stack images were acquired using a confocal laser-scanning microscope (SP5; Leica) and processed using ImageJ (rsb.info.nih.gov/ij/) to measure the area, chromocenter numbers, and RHF (*n* > 100 nuclei for each sample).

### QTL analysis and statistics

Morphometric trait values of nuclei and chromocenters (average value per nucleus) of at least 26 nuclei from at least two individual plants per RIL were used for QTL mapping. Outliers were removed from the dataset before QTL mapping was performed. This was done by excluding all values that extended beyond the 95% interval from the mean. The core marker map (Alonso-Blanco *et al.* 1998) was used for QTL analysis using the QTL-Cartographer algorithm, composite interval mapping (http://statgen.ncsu.edu/qtlcart/WQTLCart.htm), employing the “forward and backward” search method (parameters used: 10 cM window; P_in_: 0.05 P_out_: 0.05). The threshold value was determined using QTL-Cartographer by a 1000 permutation test (95% confidence interval).

Broad sense heritability (BSH) was calculated by the proportion of between-line trait variance divided by the total trait variance (using variation in a population of nuclei and chromocenters derived from a RIL as within variance and variation between the RILs as between variance). Variance was calculated by a generalized linear model with the SPSS statistical package by means of an ANOVA type 3.

The statistical programming language R (https://www.r-project.org/) was used for statistical analyses and data visualizations (R Core Team 2015). Pearson correlations and significance used for the correlation network were calculated in R. Cytoscape (http://www.cytoscape.org/) was used to visualize the correlation network of nucleus and chromocenters morphometric parameters (Shannon *et al.* 2003).

### Data availability

All (raw) data presented in this article, including nucleus and chromocenter morphometric trait values per measured nucleus and per line, all QTL profiles, additive effects, and explained variances are available in Supplemental Material, File S1.

## Results

### L*er* and Cvi-0 mesophyll cells display contrasted nucleus and chromocenter morphometric traits

**Figure 1:**
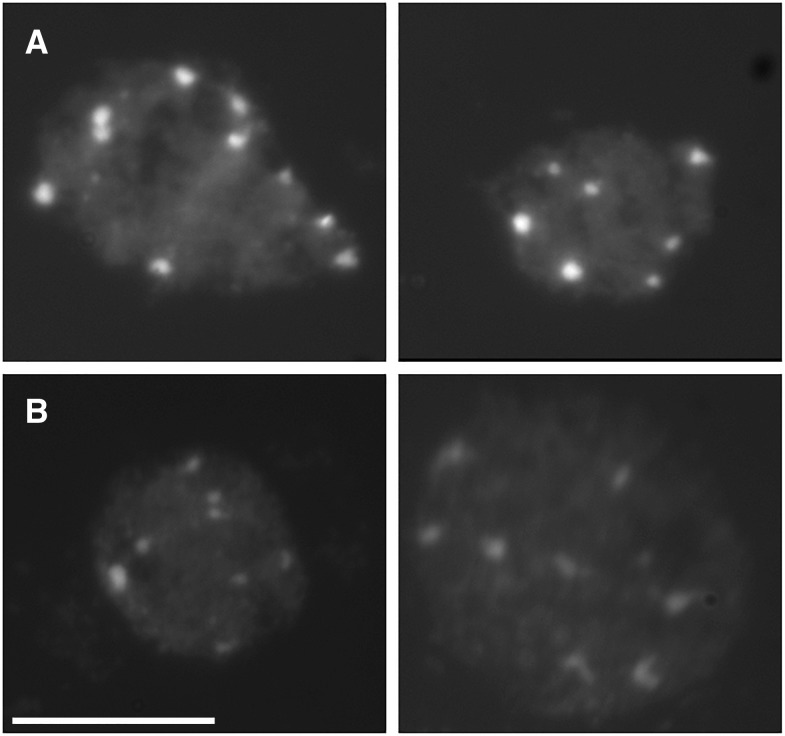
Typical images of DAPI-stained interphase leaf mesophyll cell nuclei of *Arabidopsis thaliana*. (A) The nuclei of L*er* accession shows distinct and conspicuous chromocenters, while those of (B) Cvi-0 are more diffuse and irregularly shaped. Scale bar corresponds to 5 μm.

**Figure 2:**
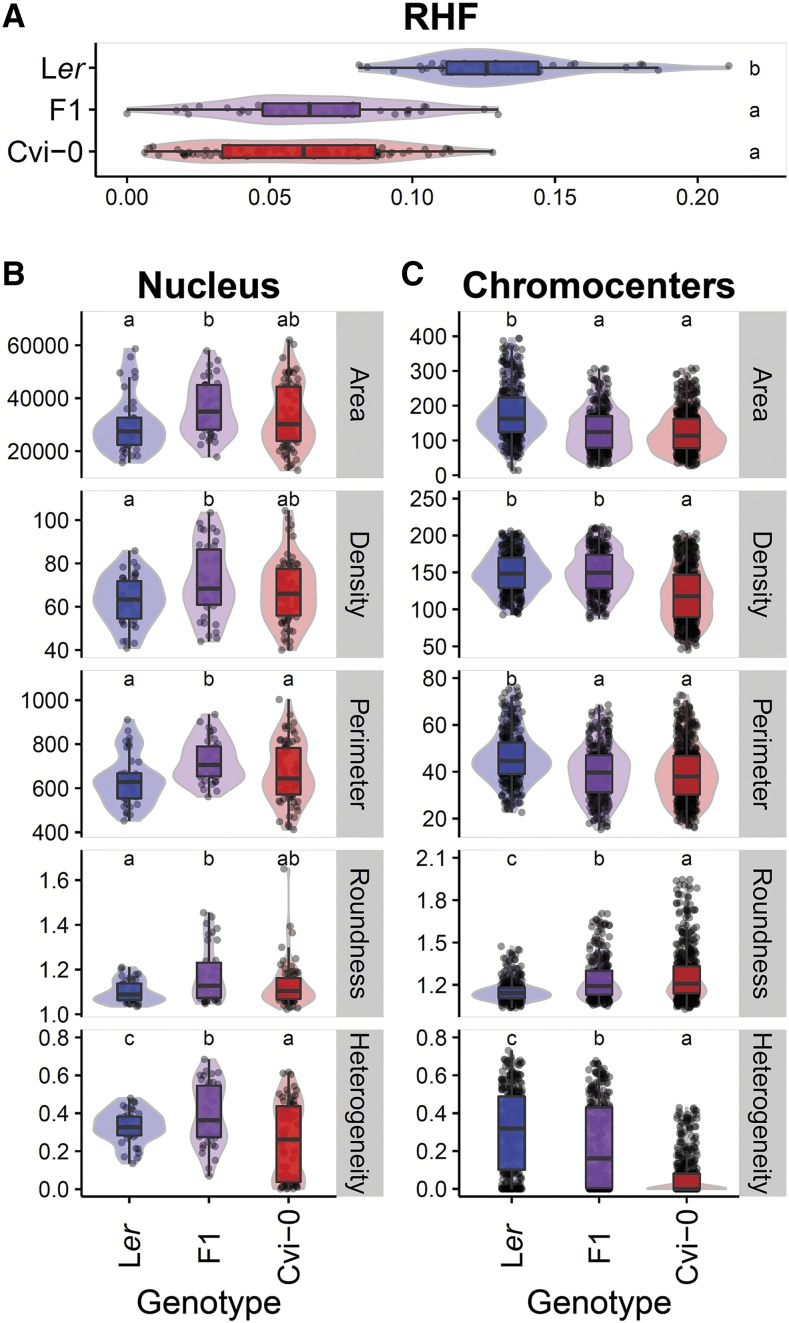
Quantification of nucleus and chromocenter morphometric parameters. (A) RHF (Tessadori *et al.* 2009), (B) morphometric parameters of nuclei, and (C) morphometric parameters of chromocenters (averaged per nucleus) of DAPI-stained interphase nuclei, of L*er* (blue), Cvi-0 (red), and F1 hybrid (purple). Boxes indicate the boundaries of the second and third quartile of the data distribution. Black bars within the boxes indicate the median and the error bars (whiskers) indicate the values in Q1 and Q4 within 1.5 times the interquartile range. Observations outside 1.5 times the interquartile range are indicated as dots. Light colored areas behind the boxes indicate sample density. Significance levels are indicated as letters above the bars and represent a two-side *t*-test assuming unequal variances. Different letters indicate significant differences (*P* < 0.01) per panel.

Under standard laboratory conditions, chromatin organization of leaf mesophyll interphase nuclei of the accession Cvi-0 greatly differs from the laboratory strain L*er* (Figure 1, A and B). The average chromocenter number per nucleus was slightly different, being 8.4 in Cvi-0, 9.3 in L*er*, and 8.5 in F1 hybrids obtained from a cross between Cvi-0 and L*er*. However more strikingly, Cvi-0 chromocenters are smaller and have an irregularly shaped appearance (Figure 1, A and B and Figure 2). Accordingly, Cvi-0 has a low RHF compared to L*er* (Figure 2A) (Tessadori *et al.* 2007b, 2009).

We quantified various morphometric traits of chromocenters and nuclei to assess which parameters underlying composite RHF contribute to low heterochromatin compaction in Cvi-0 leaf mesophyll nuclei. We measured five nuclear parameters (area, density, perimeter, roundness, and heterogeneity; see Table 1). Only heterogeneity, which refers to the uniformity of chromatin (DNA) compaction, differed significantly between Cvi-0 and L*er* (Figure 2B). Nevertheless, L*er* did differ significantly in all five measured nucleus traits from F1 hybrids (Figure 2B). Cvi-0, on the other hand, only differed significantly from the F1 hybrids in nucleus perimeter and heterogeneity (fraction of pixels that deviate >10% from the averaged intensity, hence low heterogeneity value represents high heterogeneity; Figure 2B). This indicates that Cvi-0 likely contains dominant-effect loci controlling nucleus area, density, and roundness, in agreement with the observed dominance of Cvi-0 in RHF (Tessadori *et al.* 2009). Alternatively, several positive- and negative-effect loci controlling individual nucleus morphology traits may exist in both L*er* and Cvi-0, of which the combined effect results in the observed phenotypes. The observation that the heterogeneity value of the hybrid F1 significantly extends the value of both parental lines supports this hypothesis (Figure 2B).

The absence of clear differences between Cvi-0 and L*er* in most nucleus morphometric parameters suggests that the low RHF of Cvi-0 is largely determined by differences in chromocenter morphometric traits. This is in line with the observation that next to a low RHF also the HX, a qualitative measure of chromatin compaction independent of nucleus morphology, is low in Cvi-0 compared L*er* (Tessadori *et al.* 2009). Accordingly, Cvi-0 and L*er* differed significantly in all measured morphometric chromocenter parameters (Figure 2C). Moreover, L*er* differed significantly from all parameter values from the hybrid F1, except density. In addition, Cvi-0 and F1 chromocenter density, roundness, and heterogeneity values were significantly different from each other (Figure 2C).

Especially large differences were observed in absolute values of chromocenter area and heterogeneity when comparing morphometric traits between Cvi-0 and L*er*. In other words, compared to L*er*, chromocenters of Cvi-0 are small (low area and perimeter), less round (roundness value deviating from one) and more heterogeneous (low heterogeneity value; Figure 2C). These data are in line with the visual observations that L*er* chromocenters are more condensed and conspicuous than in Cvi-0 plants (Figure 1, A and B) (Tessadori *et al.* 2009), which further suggests that the low RHF of Cvi-0 nuclei is largely assigned to small and diffuse chromocenters of Cvi-0 compared to L*er* and not directly by differences in nucleus morphology.

However, when we compared the distribution curves of nucleus area values of Cvi-0, L*er*, and F1 (Figure S1 in File S2), we observed that the fraction of very large nuclei in the measured population of Cvi-0 nuclei is significantly higher than in L*er* (*γ*^2^ test: comparing nuclei <40,000 *vs.* >40,000 pixels; *P* = 0.0184), whereas comparison of average nucleus area for the same trait and the same population of nuclei did not differ significantly (*t*-test: Cvi-0 × L*er*, *P* = 0.247; Cvi-0 × F1, *P* = 0.167; L*er* × F1, *P* = 0.0511) (Figure 2B). This shows that our mathematical approach based on averages does not fully rule out contribution of nucleus morphometric trait distributions to RHF within the population of nuclei.

### Multiple alleles underlie natural variation in nucleus and morphometric chromocenter parameters

**Figure 3:**
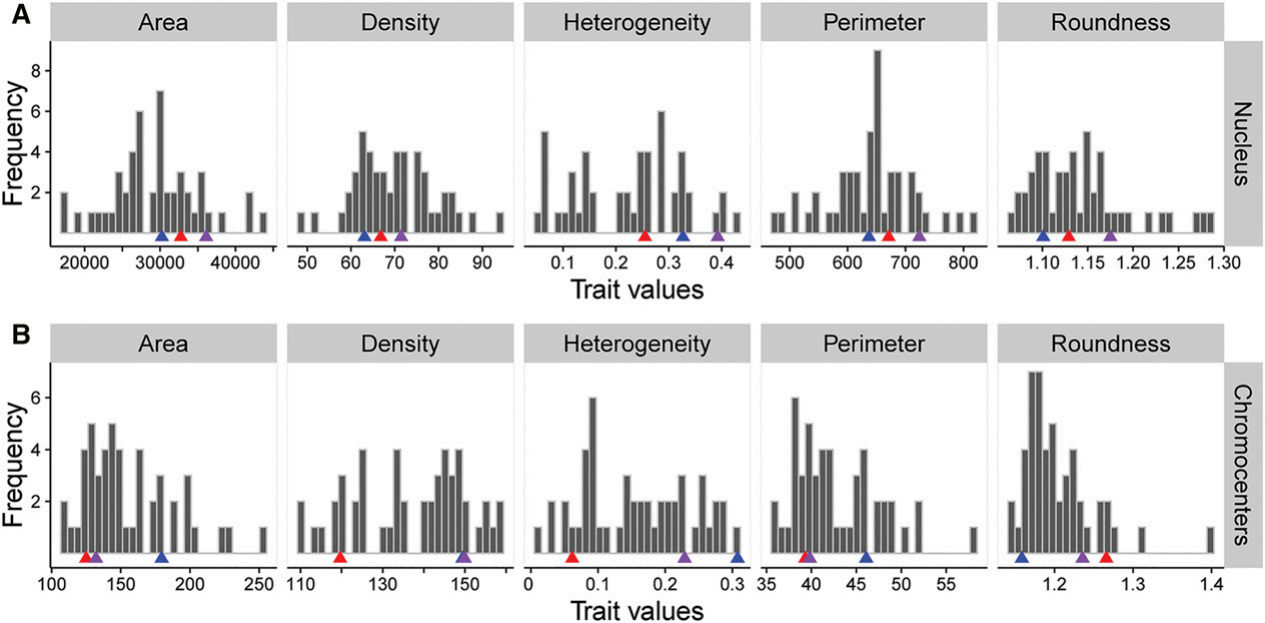
Nucleus and chromocenter trait value distributions in RILs and parental lines. Frequency distribution of trait values (in absolute occurrences/per trait, indicated by the gray bars) of (A) nucleus and (B) chromocenters morphometric parameters obtained from mesophyll interphase nuclei of 46 RILs of the Cvi-0 × L*er* population (Alonso-Blanco *et al.* 1998). Trait averages of the parental lines are indicated by red (Cvi-0) and blue (L*er*) arrows and hybrid F1 is indicated by purple arrows. Outliers beyond two times the SD from the mean per line, per trait, and tested plant genotype, were removed prior to classification. Note the occurrence of transgression for each morphometric trait.

Genetic loci contributing to segregating quantitative trait variation can be detected by QTL analysis (Alonso-Blanco *et al.* 1998, 2009; Weigel 2012). We measured morphometric parameters of nuclei and chromocenters (Table 1) in a core population of 46 RILs derived from a cross between L*er* and Cvi-0 (Alonso-Blanco *et al.* 1998) (Figure S2 in File S2), with the goal of detecting QTL for these traits. With the exception of roundness, the observed nucleus trait distributions reflected a Gaussian curve (normal distribution) (Figure 3 and Table S1 in File S2). Conversely, it was found that with the exception of density, all chromocenter traits deviated from a normal distribution. Transgression was observed for all traits, *i.e.*, the phenotypic values of RILs extend beyond those of the parental lines and the F1 hybrid (Figure 3 and Figure S2, B and C in File S2). This indicates that positive- and negative-effect loci segregate in the RIL population, and hence, each trait is influenced by interactions between multiple loci, resulting in the observed phenotype in a given RIL. The largest variation in nucleus traits was observed for heterogeneity (lowest value being 17.4% of that of the highest) and the smallest variation was observed for roundness (76.1%). The chromocenter traits show less transgression beyond the parental phenotypes, with heterogeneity (8.9%) having the largest variation and density (72.5%) the smallest variation.

**Figure 4:**
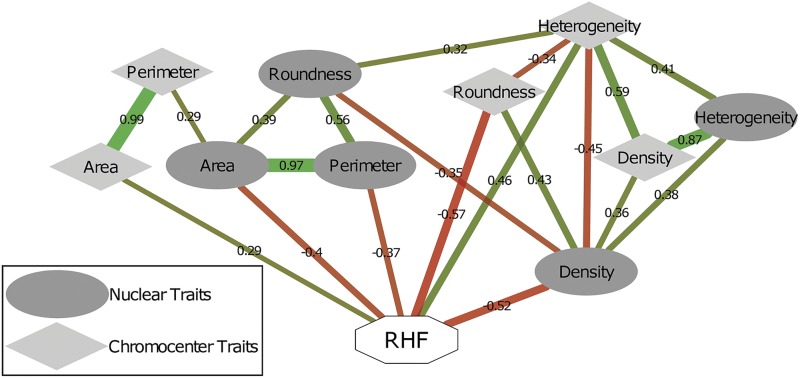
Trait correlation network of nucleus and chromocenter morphometric traits. Correlation network based on nucleus and chromocenters morphometric parameters of the tested RILs, parental lines Cvi-0 and L*er* and hybrid F1 (Figure 2). Nucleus morphometric traits are shown as circles and chromocenter morphometric traits as diamonds. Composite RHF is shown as a white octagon. Significant correlations between traits are shown as edges (see Table 2 for an overview of Pearson *R*^2^ correlation values and Table S2 in File S2 for their significance). Positive correlations are shown in green and negative in red. Color depth and line width indicates the strength of the correlation. Correlations (Pearson; *R*^2^) are shown in black.

Correlation analysis among individual traits is informative in revealing associations and/or mutual exclusion of traits. Various significant intraspecific correlations among nucleus or chromocenter morphometric values were detected (Figure 4, Table 2, and Table S2 in File S2). For instance, area and perimeter have a strong positive correlation in both the nucleus and chromocenter datasets (Figure 4, Table 2 and Table S2 in File S2). In other words, nuclei and chromocenters with a large area (volume) also tend to have a large perimeter, as expected. Nucleus area and perimeter also strongly correlates with roundness (Figure 4, Table 2 and Table S2 in File S2), which suggests limited occurrence of invaginations of the nucleus envelope (Walters *et al.* 2012) in the measured populations of nuclei. We further observed no correlation between area/perimeter and roundness for chromocenters, which points at the dynamic shape of individual chromocenters.

**Table 2:** Pearson correlations between averaged trait values obtained from the RILs, parental lines, and F1 hybrid

	RHF	Area	Density	Perimeter	Roundness
Nucleus					
Area	−0.40 (**)				
Density	−0.52 (***)	−0.04			
Perimeter	−0.37 (**)	0.97 (****)	−0.09		
Roundness	0.09	0.39 (**)	−0.35 (*)	0.56 (****)	
Heterogeneity	−0.05	0.19	0.38 (**)	0.22	0.10
Chromocenters					
Area	0.29 (*)				
Density	0.00	−0.22			
Perimeter	0.18	0.99 (****)	−0.20		
Roundness	−0.57 (****)	−0.07	0.02	0.06	
Heterogeneity	0.46 (***)	−0.04	0.59 (****)	−0.09	−0.34 (*)
	Area	Density	Perimeter	Roundness	Heterogeneity
Nucleus *vs.* Chromocenters					
Area	0.28	0.16	0.29 (*)	0.14	0.13
Density	−0.09	0.36 (*)	0.00	0.43 (**)	−0.45 (**)
Perimeter	0.18	0.23	0.19	0.14	0.21
Roundness	−0.07	0.16	−0.10	0.01	0.32 (*)
Heterogeneity	−0.12	0.87 (****)	−0.09	0.17	0.41 (**)

Correlations are shown of nucleus (top), chromocenter (middle), and nucleus *vs.* chromocenter (bottom) morphometric parameters. For chromocenter traits, the data are based on the averaged values per nucleus before the average per RIL was calculated. Significant correlations are indicated with asterisks: * *P* < 0.05; ** *P* < 0.01; *** *P* < 0.001; **** *P* < 0.0001. For significance values, see Table S2 in File S2.

Only a limited set of interspecific correlations were detected, in accordance with previous observations that chromocenter and nucleus size dynamics are regulated independently in different developmental and genetic contexts (van Zanten *et al.* 2011; Bourbousse *et al.* 2015). Of note, however, is the strong correlation (0.87) between nucleus heterogeneity and chromocenter density (Figure 4, Table 2, and Table S2 in File S2). This is in line with the diffuse appearance of heterochromatin in the nucleoplasm *vs.* highly compacted heterochromatin in chromocenters (Tessadori *et al.* 2004, 2009; Fransz and de Jong 2011). The morphometric analyses further revealed that the parameters of nucleus area, density, and perimeter and chromocenter roundness are negatively correlated (*P* < 0.01) with the RHF.

### Morphometric QTL collocate with RHF QTL

**Figure 5:**
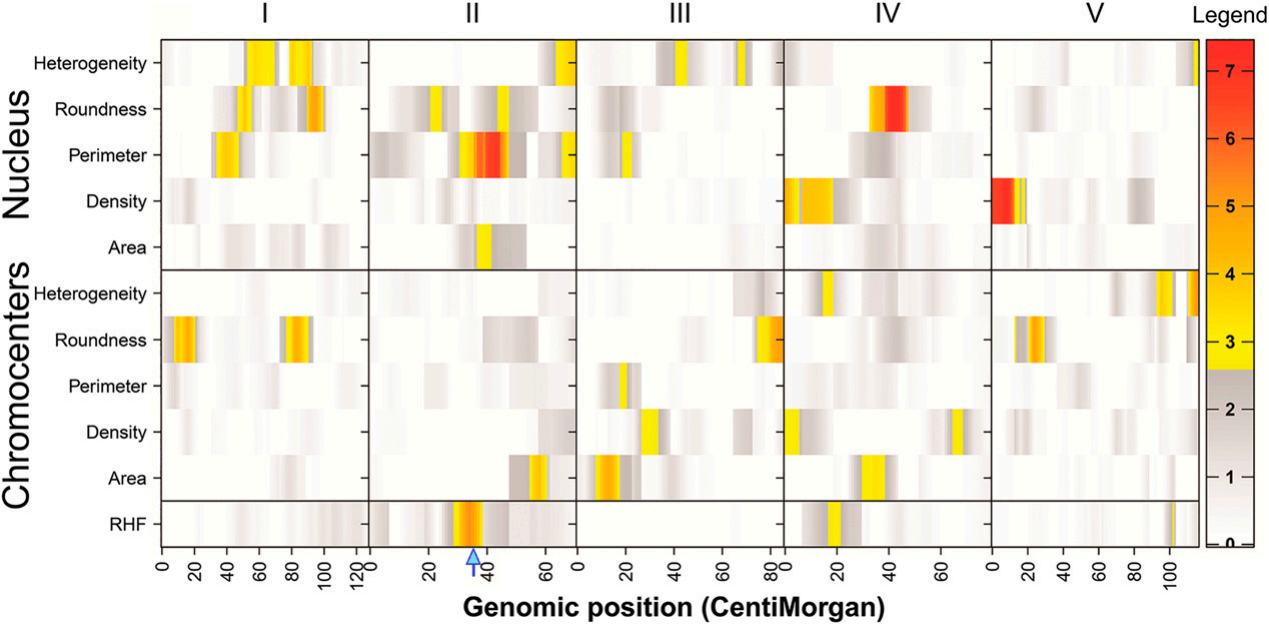
Heatmap summary of all QTL discussed in this study for nucleus and chromocenters morphometric parameters. Significance (−10 log(*p*) (LOD) scores are indicated in colors. Genome-wide significance thresholds were determined by a 1000-permutation test for each trait (Table S4 in File S2). For clarity, the LOD threshold in this figure is set at the lowest value detected in our set (2.61; density CCs, Table S4 in File S2). The lower row indicates the three QTL previously mapped for RHF (RHF2, RHF4, and RHF5) (Tessadori *et al.* 2009). See Table 3 for a numeric description of all QTL and for QTL profiles, including additive effects and QTL names, see Figure S2 in File S2. The genomic location of *PhyB* (chromosome 2, 8.14 Mb, 34.5 cM) is indicated by a blue arrow.

Calculation of BSH values (*H*^2^) indicated that between 14.1% (area) and 38.5% (heterogeneity) of the total variation observed in nucleus traits, and 10.8% (roundness) and 27.3% (heterogeneity) in chromocenter traits, can be explained by genetic variation segregating among the RILs (Table S3 in File S2). For each trait, one to five QTL (Figure 5, Figure S3 in File S2, and Table 3) could be identified above the permutation-calculated likelihood-of-odds (LOD) threshold (Table S4 in File S2). Both positive- and negative-effect QTL were detected for all traits for which multiple QTL were mapped (Figure S3 in File S2 and Table 3). This indicates that these traits are likely affected by multiple genes with contrasting allelic effects, in line with observed transgression among the RILs (Figure 3 and Figure S2B in File S2).

**Table 3:** Overview of nucleus and chromocenter morphometric trait QTL and their confirmation by NILs

	**Nucleus**	**CHR**	**Start**	**End**	**Top**	**LOD**	**ADD**	***R^2^***	**Explaining NILs**	**Explaining Borders**		**Chromocenter**	**CHR**	**Start**	**End**	**Top**	**LOD**	**ADD**	***R^2^***	**Explaining NILs**	**Explaining Borders**	
Area	**Area_Nuc_2**	**2**	**164**	**166**	**165**	**2.96**	**−2184.45**	**11.63**	**LCN2-7**	**162**	**173.2**	Area_CC_2	2	182	186	185	4.01	21.79	13.85	LCN2-7, LCN2-11	Nc	
												Area_CC_3	3	206	214	210	4.63	−22.34	15.42		Nd	
												Area_CC_4	4	313	319	317	3.33	−17.96	12.32	LCN4-3	Nc	
Density	Dens_Nuc_4-1	4	282	286	282	4.65	4.43	14.8	LCN4-2, LCN3-17	Nc		Dens_CC_3	3	224	230	224	3.11	−4.46	10.08		Nd	
	**Dens_Nuc_4-2**	**4**	**289**	**300**	**289**	**4.05**	**4.59**	**17.8**	**LCN4-2**	**290**	**297.5**	**Dens_CC_4-1**	**4**	**282**	**286**	**284**	**2.93**	**4.86**	**11.39**	**LCN3-17, LCN4-2**	**270.1**	**288.5**
	Dens_Nuc_5	5	361	375	369	7.4	−5.92	24.17		Nd		Dens_CC_4-2	4	347	350	349	2.71	5.01	11.21		Nd	
Perimeter	Per_Nuc-1	1	34	47	40	3.78	31.8	14.2		Nd		Per_CC_3	3	215	216	215	3.09	−3.07	16.31		Nd	
	**Per_nuc-2-1**	**2**	**159**	**174**	**170**	**6.76**	**−42.11**	**24.39**	**LCN2-7**	**162**	**173.2**											
	Per_nuc-2-2	2	193	197	196	3.42	31.34	13.06		Nd												
	Per_nuc_3	3	216	219	218	2.82	−31.35	12.41		Nd												
Roundness	Round_Nuc_1-1	1	49	53	51	3.83	0.03	10.31		Nd		Round_CC_1-1	1	9	19	16	4.59	−0.04	13.89		Nd	
	Round_Nuc_1-2	1	90	99	94	4.81	−0.03	14.19		Nd		Round_CC_1-2	1	80	88	83	4.48	0.04	14.66		Nd	
	Round_Nuc_2-1	2	150	150	150	3.1	0.02	8.07	LCN2-4, LCN2-5	Nc		Round_CC_3-1	3	274	274	274	3.3	−0.04	12.94	LCN3-17, LCN4-2	Nc	
	**Round_Nuc_2-2**	**2**	**173**	**173**	**173**	**3.19**	**−0.02**	**9.28**	**LCN2-7, LCN2-11**	**173.2**	**197,7**	Round_CC_3-2	3	276	282	281	4.92	−0.05	16.12	LCN3-17, LCN4-2	Nc	
	Round_Nuc_4	4	315	329	324	7.38	0.04	26.86	LCN3-17, LCN4-3	Opposite sign		Round_CC_5	5	382	389	385	4.93	−0.04	15.17	LCN5-4	Nc	
Heterogeneity	Het_Nuc-1-1	1	55	68	57	3.47	−0.04	12.79		Nd		Het_CC_4	4	298	300	300	3.1	−0.03	15.42		Nd	
	Het_Nuc-1-2	1	79	83	81	3.56	0.04	13.83		Nd		Het_CC_5_1	5	455	461	456	3.74	0.04	16.21	LCN5-16	Nc	
	Het_Nuc-1-3	1	85	92	90	3.81	0.05	15.36		Nd		Het_CC_5_2	5	472	477	477	5.21	−0.04	26.22	LCN5-16, LCN5-17	Nc	
	**Het_Nuc_2**	**2**	**192**	**197**	**196**	**3.66**	**0.04**	**13.8**	**LCN2-7, LCN2-11**	**173.2**	**197,7**											
	Het_Nuc_5	5	477	477	477	2.87	−0.04	10.93	LCN5-16, LCN5-17	Nc											
RHF	RHF2	2	158	164	161	4.86	0.014	15.7														
	RHF4	4	298	304	301	3.82	−0.01	16.4														
		5	462	464	463	3.21	0.013	15.9														

CHR indicates the chromosome number where the QTL is located; start and end indicate the borders (in cM) in-between which the QTL is located (QTL interval values above the LOD significance threshold); Top indicates the position of maximum QTL LOD value (in cM); LOD indicates the likelihood-of-difference score of the Top; ADD indicates the additive effect (L*er* allele effect compared to the population average); and R^2^ shows variance in percentages. RHF QTL that are underlined are from Tessadori *et al.* (2009). QTL that could be confirmed by NILs are indicated in bold. The confirming NILs and their introgression borders between which the significant effect of the phenotype was confirmed (in cM) are indicated. Nd indicates that the QTL was not covered by the NILs included in this study. Not confirmed (Nc) indicates that the QTL was covered by NIL introgressions, but could not be confirmed. ‘Opposite sign’ indicates that the NIL covering the QTL had an effect that was significantly different than in L*er*, but opposite of the QTL effect. See Table 4 for more details on NIL effects and QTL confirmation, and Table S5 in File S2 for introgression borders of the used NILs.

Colocation of QTL, *i.e.*, occurrence of QTL for different traits on the same genomic region, was not frequent (Figure 5, Figure S3 in File S2, and Table 3). This indicates that several allelic variants that contribute to individual morphometric traits segregate between L*er* and Cvi-0, and that individual morphometric traits have a unique molecular basis. This is in agreement with previous observations that chromocenter and nucleus size dynamics are controlled by largely independent molecular processes (van Zanten *et al.* 2011; Bourbousse *et al.* 2015). Interestingly, however, some major QTL of individual morphometric parameters do colocate with QTL for RHF that were reported before by Tessadori *et al.* (2009). For instance, RHF2 (Tessadori *et al.* 2009) colocates with the negative-effect QTL Area_Nuc_2 and Per_Nuc_2-1 reported here (Figure 5, Figure S3 in File S2, and Table 3). The positive-effect RHF QTL on chromosome 4 (RHF4) (Tessadori *et al.* 2009) colocates with the positive-effect QTL Dens_Nuc_4-2 and Het_CC-4, and the positive-effect RHF QTL on chromosome 5 (RHF5) (Tessadori *et al.* 2009) is in the vicinity of Het_CC_5-1 and Het_CC_5-2 (Figure 5, Figure S3 in File S2, and Table 3). Such colocalization could indicate that the same segregating causal allele(s) within the QTL interval is/are responsible for both the observed natural phenotypic variation in individual morphometric traits and for the composite RHF. Hence, this individual morphometric trait could directly influence the RHF value. This reasoning is supported by the observation that nucleus area, perimeter, and density variables in the RILs show a negative correlation with RHF, *i.e.* where the area, perimeter, or density of the nucleus increase, the RHF decreases (Figure 4 and Table 2). The same can be argued for chromocenter heterogeneity, which shows a significant positive correlation with RHF.

### Confirmation of the detected QTL by NIL analysis

To confirm the QTL positions and their effects and to confine the QTL intervals, we measured nucleus and chromocenter morphometric parameters in a selection of near isogenic lines (NILs) that cover and flank the QTL positions (Table 4 and Figure S4 and Table S5 in File S2). These NILs contain (small) introgressed regions of the Cvi-0 genome in an otherwise isogenic L*er* genomic background (Keurentjes *et al.* 2007) (Table S5 in File S2). If a difference in trait values is found between a given NIL and L*er*, this difference can be attributed to a causative allele segregating between L*er* and Cvi-0 located within the introgressed region.

**Table 4:** Confirmation of QTL by NILs

	Nucleus					Chromocenters				
	Area	Density	Perimeter	Roundness	Heterogeneity	Area	Density	Perimeter	Roundness	Heterogeneity
LCN 2-4	ns	D (**)	ns	ns	D	D (**)	D (**)	D (**)	ns	ns
LCN 2-5	ns	ns	ns	ns	D	D (**)	D (**)	D (**)	U (**)	ns
LCN 2-7	**u** (*)	D (**)	**U** (**)	**U** (**)	**d** (*)	U (**)	D (**)	U (**)	d (*)	ns
LCN 2-11	ns	ns	ns	**U** (**)	**d** (*)	ns	d (*)	ns	ns	U (**)
LCN 3-17	D (**)	ns	D (**)	ns	ns	D (**)	**D** (**)	D (**)	ns	ns
LCN 4-2	ns	**D** (**)	ns	U (**)	D	ns	**D** (**)	ns	D (**)	U (**)
LCN 4-3	ns	D (**)	ns	U (**)	D	ns	D (**)	ns	D (**)	U (**)
LCN 5-3	ns	ns	ns	U (**)	ns	ns	D (**)	ns	u (*)	ns
LCN 5-4	D (**)	u (*)	D (**)	ns	u (*)	D (**)	ns	D (**)	ns	d (*)
LCN 5-16	d	ns	ns	ns	ns	ns	D (**)	ns	ns	ns
LCN 5-17	D (**)	ns	D (**)	u (*)	ns	u (*)	ns	u (*)	D (**)	ns

Overview of effects of nucleus and chromocenter morphometry traits in various L*er* × Cvi-0 NILs (LCN), sorted on the occurrence of the main Cvi-0 introgression in the L*er* genetic background. U/u (up) indicates that the trait value is significantly higher in the NIL than in the L*er* parental background and D/d (down) indicates that the trait value is significantly lower than in L*er*. Trait values that are significantly different at the *P* < 0.05 level are indicated in small letters and (*). Values that are significantly different at the *P* < 0.01 level are indicated in capital letters and (**). NIL trait values that are not significantly different from L*er* are indicated as “ns.” Trait values that confirm QTL effects (Table 3) are bold.

Based on the introgression positions (Table S5 in File S2) we anticipated that the set of L*er* × Cvi-0 NIL (LCN) lines used in this study together could explain nine nucleus and eight chromocenter QTL. We were able to ratify five nucleus morphometry QTL (Area_Nuc_2, Dens_Nuc_4-2, Per_Nuc_2-1, Round_Nuc_2-2, and Het_Nuc_2), while four of them could not be confirmed (Dens_Nuc_4-1, Round_Nuc_2-1, Round_Nuc_4, and Het_Nuc_5). Several QTL could not be challenged by NIL analysis, as these QTL mapped to regions that are not covered by the Cvi-0 introgressions in the used set of NILs. This was the case for nine nucleus and seven chromocenter QTL (Table 4 and Figure S4, Table S5, and Table S6 in File S2).

Among the confirmed QTL were those that colocate with RHF2 (Area_Nuc_2 and Per_Nuc_2-1; Figure 3, Table 3, and Table S6 in File S2). These negative-effect QTL could be confirmed using LCN2-7 plants. In contrast, nuclei of LCN2-4, LCN2-5, and LCN2-11 plants did not differ significantly from L*er* in area and perimeter (Table 4 and Figure S4 and Table S6 in File S2), restricting the genomic interval explaining Area_Nuc_2 and Per_Nuc_2-1 between 162 and 173.2 cM (Table S5 in File S2). Similarly, Round_Nuc_2-2 was confirmed by LCN2-7. In contrast, nucleus shapes from LCN2-4 and LCN2-5 plants were not significantly different from L*er* (Figure S4 and Table S6 in File S2). However, like LCN2-7, LCN2-11 also confirmed the QTL. Therefore, the causal allele can be assigned to the region between 173.2 and 197.7 cM, and thus is probably different from the allele that explains Area_Nuc_2 and Per_Nuc_2-1.

The positive-effect QTL Het_Nuc_2 was explained by LCN2-7 and LCN2-11, as in Round_Nuc_2-2. However, LCN2-4 and LCN2-5 also had a significantly lower heterogeneity (Table 4 and Figure S4 and Table S6 in File S2), whereas no heterogeneity QTL mapped to the genomic regions introgressed in LCN2-4 and LCN2-5 (Figure 5, Figure S3 in File S2). This indicates that at least two alleles with positive effect (L*er*) on nucleus heterogeneity are present on chromosome 2. These could be the same alleles that explain Area_Nuc_2 and/or Per_Nuc_2-1 and Round_Nuc_2-2.

The positive-effect QTL Dens_Nuc_4-1 is covered by introgressions of LCN3-17 and LCN4-2. Both introgressions have the same borders (Table S5 in File S2), but only LCN4-2 (and not LCN3-17) had a lower density than L*er* (Figure S4 and Table S6 in File S2). Therefore, this QTL could only be partially confirmed. Nevertheless, the significantly lower density of LCN4-2 compared to L*er* did confirm Dens_Nuc_4-2.

Of the chromocenter morphometric QTL (Figure 5 and Table 3), Dens_CC_4-1 could be confirmed. Dens_CC_4-1 is a positive-effect QTL and, accordingly, both the LCN3-17 and LCN4-2 NILs indeed had a significantly lower density than L*er* (Table 4 and Figure S4 and Table S6 in File S2).

Additional QTL that were not detected by the initial QTL mapping were detected for all morphometric parameters by directly comparing the NIL introgression lines to the wild-type L*er* background (Table 4 and Figure S4 and Table S6 in File S2). This underlines the polygenic and highly complex genetic architecture of morphometric traits of both nucleus and chromocenters.

### PhyB is a negative regulator of nucleus size

In our previous work (Tessadori *et al.,* 2009), we demonstrated that RHF2 can be explained by natural genetic variation in the photo/thermoreceptor *PhyB* (Koornneef *et al.* 1980; Jung *et al.* 2016; Legris *et al.* 2016). *PhyB* is located on position 160.5 cM on the L*er* genetic map (www.Arabidopsis.org) and Cvi-0 and L*er* *PhyB* alleles differ at multiple nucleotides along its coding sequence (Filiault *et al.* 2008). The *PhyB* locus is covered by the QTL LOD confidence interval of Per_Nuc_2-1 (159–174 cM), but is just outside the confidence interval of Area_Nuc_2 (164–166 cM). However, only a small part of the latter QTL meets the significance threshold and the *PhyB* locus is part of the broader area QTL in this genomic region (see Figure S3 in File S2). The *PhyB* locus is covered by the introgression regions of LCN2-4, LCN2-5, and most likely, LCN2-7 (Table S5 in File S2). Although Area_Nuc_2 and Per_Nuc_2-1 could not be confirmed in the LCN2-4 and LCN2-5 NILs, the nucleus phenotypes of LCN2-7 did confirm these QTL (Table 4 and Figure S4 and Table S6 in File S2).

**Figure 6:**
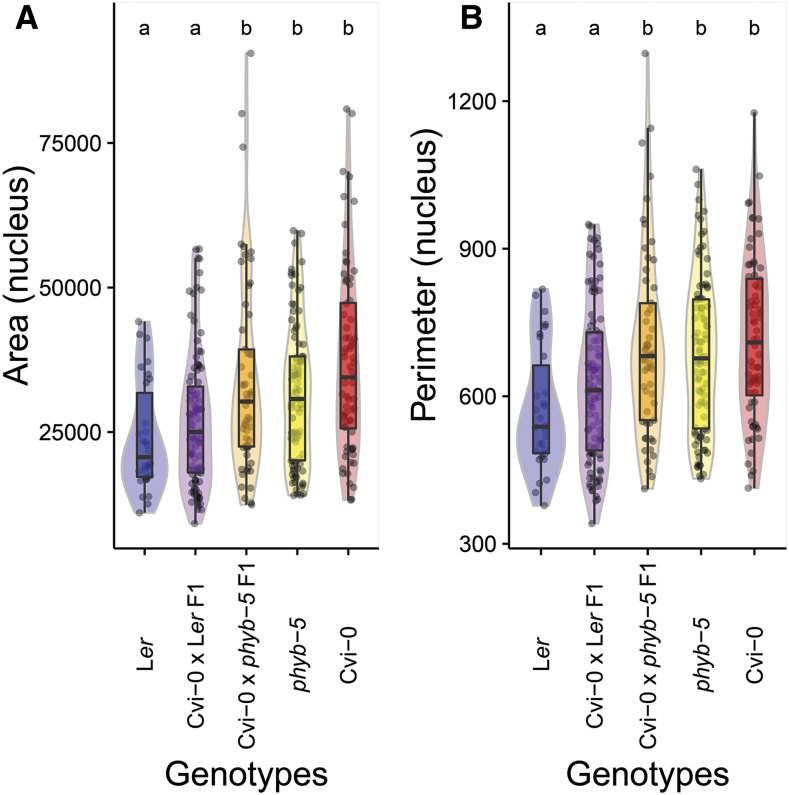
Mutant analyses and genetic complementation tests confirm the PhyB effect on nucleus area and perimeter. (A) nucleus area and (B) nucleus perimeter of parental lines L*er* (blue) and Cvi-0 (red), mutant *phyb-5* (yellow) and F1 hybrids derived from crosses: Cvi-0 × L*er* (purple) and Cvi-0 × *phyb-5* (orange). Boxes indicate the boundaries of the second and third quartile of the data distribution. Black horizontal bars within the boxes indicate the median and the error bars (whiskers) indicate the values in Q1 and Q4 within 1.5 times the interquartile range. Individual observations are shown as black dots. Light colored areas behind the boxes indicate sample density. Significance levels are indicated as letters above the bars and represent a two-side *t*-test assuming unequal variances. Different letters indicate significant differences (*P* < 0.01) per panel.

We directly tested whether *PhyB* affects nucleus size using mutant analyses and genetic complementation tests. In this experiment, Cvi-0 nuclei were significantly bigger (area and perimeter) than L*er* nuclei (Figure 6). Interestingly, mesophyll nuclei isolated from *phyb-5* mutant plants (Koornneef *et al.* 1980) also displayed significantly higher area and perimeter values than the corresponding L*er* wild-type background, reaching statistically similar sizes as Cvi-0. These results indicate that *PhyB* is a negative regulator of nucleus size in L*er*.

To directly test whether natural variation in *PhyB* could be responsible for the Area_Nuc_2 and Per_Nuc_2-1 QTL and possibly underlies nucleus size differences between L*er* and Cvi-0, we performed genetic complementation tests (Mackay 2001; Borevitz and Nordborg 2003). Hybrid F1 progeny of a cross between L*er* and Cvi-0 exhibited a similar nucleus size (area and perimeter) than wild-type L*er*, *i.e.* significantly smaller than Cvi-0 (Figure 6). This indicates that L*er* carries a dominant locus that can complement the large-nucleus phenotype of Cvi-0. In contrast, nucleus size of the F1 progeny from the Cvi-0 × *phyb-5* cross was similar to Cvi-0. Accordingly, Cvi-0 × *phyb-5* F1 hybrids did differ significantly from L*er* (Figure 6). We therefore conclude that a functional L*er* *PhyB* allele is required for the small nucleus size in the L*er* × Cvi-0 cross. Taken together, these observations show that *PhyB* is a negative regulator of nucleus size and that natural variation in *PhyB* most likely underlies the Area_Nuc_2 and Per_Nuc_2-1 QTL.

**Figure 7:**
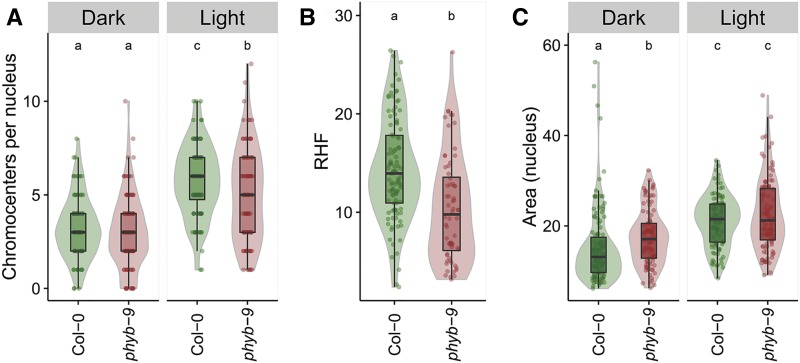
PhyB control of nucleus size and chromatin compaction dynamics during de-etiolation. (A) Number of chromocenters per nucleus, (B) RHF, and (C) nucleus area of Col-0 wild type (green) and *phyb-9* mutants (red) in cotyledon nuclei of seedlings grown in darkness (Dark) or shifted to light for 24 hr (Light). Boxes indicate the boundaries of the second and third quartile of the data distributions. Black horizontal bars within the boxes indicate the median and the error bars (whiskers) indicate the values in Q1 and Q4 within 1.5 times the interquartile range. Individual observations are shown as dots. Light colored areas behind the boxes indicate sample density. Significance levels are indicated as letters above the bars and represent a two-side *t*-test assuming unequal variances. Different letters indicate significant differences (*P* < 0.05) per panel.

To independently confirm the negative effect of *PhyB* on nucleus size, we tested the influence of the *phyb-9* mutation in a Col-0 genetic background on the dynamic increase of both nucleus size and chromocenter condensation during cotyledon de-etiolation (Bourbousse *et al.* 2015). In dark-grown seedlings, no difference was found in the number of chromocenters in cotyledon nuclei of *phyb-9* and wild-type Col-0. Exposing the etiolated seedling to white light for 24-hr induced chromocenter formation in the wild type as expected (Figure 7A and Figure S5 in File S2), but the increase in the number of chromocenters per nucleus was significantly lower in the *phyb-9* mutant (Figure 7A). This indicates that PhyB is a positive regulator of chromocenter condensation dynamics under white light conditions. This observation is in accordance with previous data showing that the HX of both *phyb-9* (in Col-0) and *phyb-5* (in L*er*) is lower than their corresponding wild types and is consistent with the observation that *phyb-9* plants display a reduced RHF compared to wild type when grown under standard light conditions (Tessadori *et al.* 2009) (Figure 7B). Interestingly, nuclei of dark-grown *phyb-9* cotyledons were significantly larger than those of Col-0 (area; Figure 7C). These data confirm that *PhyB* is a negative regulator of nucleus size (Figure 6 and Figure 7C) independently of its effect on chromatin compaction, as no differences in chromocenters are visible at this stage between the wild-type and *phyb-9* mutant nuclei. Exposure to 24-hr light, however, led to a similar increase in nucleus size in both Col-0 and *phyb-9* (Figure 7C). This suggests that *PhyB* effects on nucleus size in dark-grown seedlings is overruled by the 24-hr light exposure (Figure 7C) or that expression of the *phyb* mutant effect on nucleus size requires >24 hr of light exposure.

## Discussion

### Dissection of composite RHF and HX in distinct morphometric parameters provides comprehensive information on chromatin dynamics

The contribution of chromatin organization to nuclear structure is a topic of increasing interest (Liu and Weigel 2015; Poulet *et al.* 2017). The clear separation between microscopic appearance of euchromatin and heterochromatin makes *Arabidopsis* a system particularly well suited to study chromatin organization and dynamics (van Zanten *et al.* 2012, 2013; Probst and Mittelsten Scheid 2015; Perrella and Kaiserli 2016).

Quantification of HX and/or the RHF has been extensively used in studies on dynamics in chromatin compaction in relation to development (Mathieu *et al.* 2003; Tessadori *et al.* 2004, 2007a,b; van Zanten *et al.* 2011; Bourbousse *et al.* 2015) and response to biotic (Pavet *et al.* 2006) and abiotic stimuli (Tessadori *et al.* 2009; Pecinka *et al.* 2010; van Zanten *et al.* 2010, 2012, 2013; Wang *et al.* 2013; Bourbousse *et al.* 2015). Despite the extensive use of RHF and HX, the exact molecular determinants and the functional significance of heterochromatin condensation changes in *A. thaliana* remain largely unclear. A reason for this is that the RHF and HX values provide only partial information about the mechanisms underlying chromocenter condensation, because various (and possibly unrelated) primary parameters, such as chromocenter number, nucleus size, and chromocenter size, contribute to the composite RHF. Complementary analyses that uncover hidden information provided by other morphometric parameters were required to better understand chromatin compaction controls. For example, an increase in RHF could either be the result of chromocenter enlargement or densification with maintenance of nucleus size or reciprocally result from a decrease in nucleus size or density with maintenance of chromocenter size. Hence, similar changes in composite RHF could be caused by distinct molecular mechanisms.

Gaining full understanding of the biological function of chromatin (de)condensation and the contributing molecular factors, requires an experimental genetic system in which morphometric parameters underlying chromatin organization can be independently quantified. In this work, we exploited the natural existence of genetic variation in nucleus and chromocenter morphometric parameters to provide such new fundamental insights in the molecular genetic basis of chromocenter formation and/or maintenance. Our morphometric analyses unveiled that nuclear area, density, and perimeter, as well as chromocenter roundness and heterogeneity correlate with the RHF. This is not the case for chromocenter density and the correlation between RHF and chromocenter area is low. This suggests that condensation of heterochromatin regions and overall chromocenter organization might not be directly linked.

Of note is the apparent distinction of phenotypes in nuclear heterogeneity, which seems to fall apart into three distinguishable classes (Figure 3). This illustrates how individual morphometric traits could be informative to understand the dynamics of chromocenter compaction. Although the distribution of phenotypes of this trait still has a Gaussian distribution (Table S1 in File S2), we speculate that the distinguishable classes may reflect the three chromatin compaction types observed in a population of mesophyll nuclei; from type 1 containing conspicuous chromocenters to type 3 containing relaxed heterochromatin (Tessadori *et al.* 2009).

### Detecting QTL for nucleus and chromocenter morphometry

In general, the heritability of morphometric traits of individual chromocenters was much lower than when averaged per nucleus (Table S3 in File S2). This is indicative for a general control mechanism that simultaneously determines the chromocenter state in a particular nucleus. This observation, however, does not rule out that morphometry of particular chromocenters is individually regulated.

For each individually assessed morphometric trait one or more QTL could be mapped (Figure 5), highlighting the highly complex genetic basis of chromatin organization of *Arabidopsis* nuclei. This indicates that several molecular regulators contribute to heterochromatin condensation levels. Several QTL covered by the introgression borders of the NILs were not confirmed. Moreover, additional QTL were identified by NILs that were not detected in QTL analysis. The existence of more QTL in NILs than in RILs has been repeatedly reported (Keurentjes *et al.* 2007; Green *et al.* 2013; Snoek *et al.* 2013, 2014; Stastna *et al.* 2015). This can be caused by closely linked QTL, which are hard to detect in the RILs, or by complex interactions between QTL, for which the model “to-test” is difficult to predict. It should also be noted that our QTL detection is based on a moderately small core set of 46 RILs. A consequence of a small population size is that statistical power of QTL detection is relatively limited, *i.e.*, only strong QTL are detected. It is therefore possible that these population sizes preclude the detection of small-effect QTL, which appear in the NILs. Regardless of the reason, the presence of such extra QTL further underlines that the genetic architecture of both nucleus and chromocenter morphometric traits is polygenic and involves highly complex genetic interactions. The underlying molecular components and effects on gene expression could be further studied using colocating expression QTL (Nijveen *et al.* 2016). Taken together, our study unveils the existence of multiple loci regulating specific nucleus and chromocenter morphometric traits that together contribute to heterochromatin organization. Therefore, this work provides an experimental toolbox to identify novel genetic factors and molecular mechanisms underlying changes in chromatin compaction induced during development or in response to the environment.

### PhyB is a negative regulator of nucleus size

Much to our surprise, only limited genomic overlap (colocation) was detected among the identified QTL intervals. Though, the morphometry QTL Dens_Nuc_4-2 and Het_CC_4 colocate with the previously identified RHF4 (Figure 5, Figure S3 in File S2, and Table 3). This RHF4 could be explained by natural variation in HISTONE DEACETYLASE 6 (HDA6) (Tessadori *et al.* 2009). This suggests that also nucleus density and heterogeneity may be under the control of HDA6.

Another notable exception was the overlap between QTL for nucleus area and perimeter (Area_Nuc_2 and Per_Nuc_2-1; Figure 5, Figure S3 in File S2) with RHF2 (Tessadori *et al.* 2009), which results from natural variation in the PhyB nucleotide sequence (Tessadori *et al.* 2009). Using mutant analyses and genetic complementation tests, we were able to show that, in addition to its influence on heterochromatin organization, PhyB is also a negative regulator of nucleus size of mesophyll leaf cells (Figure 6) and in young Col-0 seedlings (Figure 7).

A likely cause of the limited overlap in QTL positions is that dynamic regulation of individual morphometric phenotypes not necessarily cooccur. This is in accordance with our previous work showing that changes in chromatin compaction and changes in nucleus size are developmentally and genetically uncoupled (van Zanten *et al.* 2011; Bourbousse *et al.* 2015). Yet they may share common signaling components or molecular determinants to cooperatively regulate nucleus reorganization events. Indeed, our genetic study of natural variation unveiled a clear correlation between nucleus size (area and perimeter) and RHF values (Figure 4 and Table 2). Hence, distinct processes such as endoreduplication and activity of chromatin remodeling proteins, at different developmental stages or in response to environmental cues, might converge to allow coordination of nucleus size and heterochromatin organization. From our observations presented here and previous work, we propose that PhyB-mediated control of nucleus size is at least in part responsible for the natural variation in chromatin condensation levels among *Arabidopsis* accessions (Tessadori *et al.* 2009).

We found that PhyB has a negative effect on nucleus size of rosette leaves grown in growth cabinets (Figure 6) or in a greenhouse (Figure 3). This is also the case in cotyledon nuclei of 5-d-old etiolated seedlings, but not upon exposing them to light for 24 hr (Figure 7C). This might directly relate to *phyB* mutant plants having defective capacity for rapid or efficient heterochromatin compaction. Light-mediated changes in chromatin compaction occur notoriously slow, however. For instance, maximum chromocenter decondensation is only reached 4 d after shifting plants to low light intensity treatment (van Zanten *et al.* 2010) and, vice versa, chromocenter formation is not completed upon 24 hr of de-etiolation (Bourbousse *et al.* 2015). The lack of a *phyB*-dependent effect on nucleus size after a relatively short exposure to light therefore could result from the early time point (24 hr), at which the phenotypic effects linked to PhyB photoperception are not yet saturated. Accordingly, chromocenter number in *phyb-9* mutants after 24 hr of light lies in-between those of dark- and light-grown Col-0 seedlings (Figure 7A). Future studies should address the precise light-dependent and temporal components of PhyB-mediated nucleus size control and dynamic chromatin compaction.

Interestingly, high temperature also induces severe chromocenter decondensation (Pecinka *et al.* 2010; Wang *et al.* 2013), a second chromatin decompaction process in which Phytochromes, acting both as a photo- and thermoreceptor, could have a role. Obtaining dynamic data under different environments at various developmental stages could be used to develop integrated computational models parameterized by experimentally determined values of individual nucleus and chromocenter morphometric traits and their correlations. Such modeling would be helpful to further elucidate the molecular mechanisms behind chromatin compaction dynamics.

## Supplementary Material

Supplemental material is available online at www.g3journal.org/lookup/suppl/doi:10.1534/g3.117.043539/-/DC1.

## References

[bib1] Alonso-Blanco C. Peeters A. J. M. Koornneef M. Lister C. Dean C., 1998 Development of an AFLP based linkage map of L*er*, Col and Cvi *Arabidopsis* *thaliana* ecotypes and construction of a L*er*/Cvi recombinant inbred line population. Plant J. 14: 259–271. 9628021 10.1046/j.1365-313x.1998.00115.x

[bib2] Alonso-Blanco C. Aarts M. G. M. Bentsink L. Keurentjes J. J. B. Reymond M., 2009 What has natural variation taught us about plant development, physiology, and adaptation? Plant Cell 21: 1877–1896. 19574434 10.1105/tpc.109.068114 PMC2729614

[bib3] Barneche F. Malapeira J. Mas P., 2014 The impact of chromatin dynamics on plant light responses and circadian clock function. J. Exp. Bot. 65: 2895–2913. 24520020 10.1093/jxb/eru011

[bib4] Borevitz J. O. Nordborg M., 2003 The impact of genomics on the study of natural variation in *Arabidopsis*. Plant Physiol. 132: 718–725. 12805600 10.1104/pp.103.023549 PMC523862

[bib5] Bourbousse C. Mestiri I. Zabulon G. Bourge M. Formiggini F., 2015 Light signaling controls nuclear architecture reorganization during seedling establishment. Proc. Natl. Acad. Sci. USA 112: E2836–E2844. 25964332 10.1073/pnas.1503512112 PMC4450433

[bib6] Brown S. W., 1966 Heterochromatin. Science 151: 417. 5322971 10.1126/science.151.3709.417

[bib7] Chandrasekhara C. Mohannath G. Blevins T. Pontvianne F. Pikaard C. S., 2016 Chromosome-specific NOR inactivation explains selective rRNA gene silencing and dosage control in *Arabidopsis*. Genes Dev. 30: 177–190. 26744421 10.1101/gad.273755.115 PMC4719308

[bib8] Filiault D. L. Wessinger C. A. Dinneny J. R. Lutes J. Borevitz J. O., 2008 Amino acid polymorphisms in *Arabidopsis* phytochrome B cause differential responses to light. Proc. Natl. Acad. Sci. USA 105: 3157–3162. 18287016 10.1073/pnas.0712174105 PMC2268601

[bib9] Fransz P. de Jong H., 2011 From nucleosome to chromosome: a dynamic organization of genetic information. Plant J. 66: 4–17. 21443619 10.1111/j.1365-313X.2011.04526.x

[bib10] Fransz P. de Jong J. H. Lysak M. Castiglione M. R. Schubert I., 2002 Interphase chromosomes in *Arabidopsis* are organized as well defined chromocenters from which euchromatin loops emanate. Proc. Natl. Acad. Sci. USA 99: 14584–14589. 12384572 10.1073/pnas.212325299 PMC137926

[bib11] Fransz P. ten Hoopen R. Tessadori F., 2006 Composition and formation of heterochromatin in *Arabidopsis* *thaliana*. Chromosome Res. 14: 71–82. 16506097 10.1007/s10577-005-1022-5

[bib12] Green J. W. M. Snoek L. B. Kammenga J. E. Harvey S. C., 2013 Genetic mapping of variation in dauer larvae development in growing populations of *Caenorhabditis elegans*. Heredity 111: 306–313. 23715016 10.1038/hdy.2013.50 PMC3807260

[bib13] Heitz E., 1928 Das heterochromatin der moose. Jahrb Wiss Bot 69: 762–818.

[bib14] Heitz E., 1929 Heterochromatin, chromocentren, chromomeren. Ber Bot. Ges 47: 274–284.

[bib15] Jung J.-H. Domijan M. Klose C. Biswas S. Ezer D., 2016 Phytochromes function as thermosensors in *Arabidopsis*. Science 354: 886–889. 27789797 10.1126/science.aaf6005

[bib16] Keurentjes J. J. B. Bentsink L. Alonso-Blanco C. Hanhart C. J. Blankestijn-De Vries H., 2007 Development of a near-isogenic line population of *Arabidopsis* *thaliana* and comparison of mapping power with a recombinant inbred line population. Genetics 175: 891. 17179089 10.1534/genetics.106.066423 PMC1800614

[bib17] Koornneef M. Rolff E. Spruit C. J. P., 1980 Genetic control of light-inhibited hypocotyl elongation in *Arabidopsis* *thaliana* (L.) Heynh. Z. Pflanzenphysiol. 100: 147–160.

[bib18] Legris M. Klose C. Burgie E. S. Costigliolo C. Neme M., 2016 Phytochrome B integrates light and temperature signals in *Arabidopsis*. Science 18: 897–900. 10.1126/science.aaf5656 27789798

[bib19] Liu C. Weigel D., 2015 Chromatin in 3D: progress and prospects for plants. Genome Biol. 16: 170. 26294115 10.1186/s13059-015-0738-6 PMC4546174

[bib20] Mackay T. F. C., 2001 The genetic architecture of quantitative traits. Annu. Rev. Genet. 35: 303–339. 11700286 10.1146/annurev.genet.35.102401.090633

[bib21] Mathieu O. Jasencakova Z. Vaillant I. Gendrel A.-V. Colot V., 2003 Changes in 5S rDNA chromatin organization and transcription during heterochromatin establishment in *Arabidopsis*. Plant Cell 15: 2929–2939. 14630972 10.1105/tpc.017467 PMC282831

[bib22] Nijveen H. Ligterink W. Keurentjes J. J. B. Loudet O. Long J., 2016 AraQTL - workbench and archive for systems genetics in *Arabidopsis* *thaliana*. Plant J. 89: 1225–1235. 10.1111/tpj.13457 27995664

[bib23] Pavet V. Quintero C. Cecchini N. M. Rosa A. L. Alvarez M. E., 2006 *Arabidopsis* displays centromeric DNAhypomethylation and cytological alterations of heterochromatin upon attack by *Pseudomonas syringae*. Mol. Plant Microbe Interact. 19: 577–587. 16776291 10.1094/MPMI-19-0577

[bib24] Pavlova P. Tessadori F. de Jong H. J. Fransz P., 2010 Immunocytological analysis of chromatin in isolated nuclei. Methods Mol. Biol. 655: 413–432. 20734277 10.1007/978-1-60761-765-5_28

[bib25] Pecinka A. Dinh H. Q. Baubec T. Rosa M. Lettner N., 2010 Epigenetic regulation of repetitive elements is attenuated by prolonged heat stress in *Arabidopsis*. Plant Cell 22: 3118–3129. 20876829 10.1105/tpc.110.078493 PMC2965555

[bib26] Perrella G. Kaiserli E., 2016 Light behind the curtain: photoregulation of nuclear architecture and chromatin dynamics in plants. New Phytol. 212: 908–919. 27813089 10.1111/nph.14269 PMC5111779

[bib27] Poulet A. Duc C. Voisin M. Desset S. Tutois S., 2017 The LINC complex contributes to heterochromatin organisation and transcriptional gene silencing in plants. J. Cell Sci. 130: 590–601. 28049722 10.1242/jcs.194712

[bib28] Probst A. V. Mittelsten Scheid O., 2015 Stress-induced structural changes in plant chromatin. Cell Signal. Gene Regul. 27: 8–16. 10.1016/j.pbi.2015.05.011 26042538

[bib29] R Core Team, 2015 *R: A Language and Environment for Statistical Computing* R Foundation for Statistical Computing, Vienna.

[bib30] Reed J. W. Nagpal P. Poole D. S. Furuya M. Chory J., 1993 Mutations in the gene for the red/far-red light receptor phytochrome B alter cell elongation and physiological responses throughout *Arabidopsis* development. Plant Cell 5: 147–157. 8453299 10.1105/tpc.5.2.147 PMC160258

[bib31] Schubert I. Fransz P. F. Fuchs J. de Jong J. H., 2001 Chromosome painting in plants. Methods Cell Sci. 23: 57–69. 11741144

[bib32] Shannon P. Markiel A. Ozier O. Baliga N. S. Wang J. T., 2003 Cytoscape: a software environment for integrated models of biomolecular interaction networks. Genome Res. 13: 2498–2504. 14597658 10.1101/gr.1239303 PMC403769

[bib33] Snoek L. B. Terpstra I. Dekter R. Van den Ackerveken G. Peeters A. J. M., 2013 Genetical genomics reveals large scale genotype-by-environment interactions in *Arabidopsis* *thaliana*. Front. Genet. 3: 317. 23335938 10.3389/fgene.2012.00317 PMC3541481

[bib34] Snoek L. B. Orbidans H. E. Stastna J. J. Aartse A. Rodriguez M., 2014 Widespread genomic incompatibilities in *Caenorhabditis elegans*. G3 4: 1813–1823. 25128438 10.1534/g3.114.013151 PMC4199689

[bib35] Soppe W. J. J. Jasencakova Z. Houben A. Kakutani T. Meister A., 2002 DNA methylation controls histone H3 lysine 9 methylation and heterochromatin assembly in *Arabidopsis*. EMBO J. 21: 6549. 12456661 10.1093/emboj/cdf657 PMC136960

[bib36] Stastna J. J. Snoek L. B. Kammenga J. E. Harvey S. C., 2015 Genotype-dependent lifespan effects in peptone deprived *Caenorhabditis elegans*. Sci. Rep. 5: 16259. 26539794 10.1038/srep16259 PMC4634109

[bib37] Tessadori F. van Driel R. Fransz P., 2004 Cytogenetics as a tool to study gene regulation. Trends Plant Sci. 9: 147–153. 15003238 10.1016/j.tplants.2004.01.008

[bib38] Tessadori F. Chupeau M.-C. Chupeau Y. Knip M. Germann S., 2007 a Large-scale dissociation and sequential reassembly of pericentric heterochromatin in dedifferentiated *Arabidopsis*. J. Cell Sci. 120: 1200. 17376962 10.1242/jcs.000026

[bib39] Tessadori F. Schulkes R. K. van Driel R. Fransz P., 2007 b Light-regulated large-scale reorganization of chromatin during the floral transition in *Arabidopsis*. Plant J. 50: 848–857. 17470059 10.1111/j.1365-313X.2007.03093.x

[bib40] Tessadori F. van Zanten M. Pavlova P. Clifton R. Pontvianne F., 2009 *PHYTOCHROME B* and *HISTONE DEACETYLASE 6* control light-induced chromatin compaction in *Arabidopsis* *thaliana*. PLoS Genet. 5: e1000638. 19730687 10.1371/journal.pgen.1000638 PMC2728481

[bib41] van Zanten M. Tessadori F. McLoughlin F. Smith R. Millenaar F. F., 2010 Photoreceptors CRYTOCHROME2 and Phytochrome B control chromatin compaction in *Arabidopsis*. Plant Physiol. 154: 1686–1696. 20935177 10.1104/pp.110.164616 PMC2996035

[bib42] van Zanten M. Koini M. A. Geyer R. Liu Y. Brambilla V., 2011 Seed maturation in *Arabidopsis* *thaliana* is characterized by nuclear size reduction and increased chromatin condensation. Proc. Natl. Acad. Sci. USA 108: 20219–20224. 22123962 10.1073/pnas.1117726108 PMC3250172

[bib43] van Zanten M. Tessadori F. Peeters A. J. Fransz P., 2012 Shedding light on large-scale chromatin reorganization in *Arabidopsis* *thaliana*. Mol. Plant 5: 583–590. 22528207 10.1093/mp/sss030

[bib44] van Zanten M. Tessadori F. Peeters A. J. M. Fransz P., 2013 Environment-induced chromatin reorganisation and plant acclimation, pp. 21–40 in *Epigenetic Memory and Control in Plants: Signaling and Communication in Plants*, edited by Grafi G. Ohad N. Springer, Berlin, Heidelberg.

[bib45] Walters A. D. Bommakanti A. Cohen-Fix O., 2012 Shaping the nucleus: factors and forces. J. Cell. Biochem. 113: 2813–2821. 22566057 10.1002/jcb.24178 PMC3471212

[bib46] Wang L.-C. Wu J.-R. Chang W.-L. Yeh C.-H. Ke Y.-T., 2013 *Arabidopsis* HIT4 encodes a novel chromocentre-localized protein involved in the heat reactivation of transcriptionally silent loci and is essential for heat tolerance in plants. J. Exp. Bot. 64: 1689–1701. 23408827 10.1093/jxb/ert030

[bib47] Weigel D., 2012 Natural variation in *Arabidopsis*: from molecular genetics to ecological genomics. Plant Physiol. 158: 2–22. 22147517 10.1104/pp.111.189845 PMC3252104

[bib48] Yelagandula R. Stroud H. Holec S. Zhou K. Feng S., 2014 The histone variant H2A.W defines heterochromatin and promotes chromatin condensation in *Arabidopsis*. Cell 158: 98–109. 24995981 10.1016/j.cell.2014.06.006 PMC4671829

