# Supplementary material for: Genetic Dissection of Morphometric Traits Reveals That Phytochrome B Affects Nucleus Size and Heterochromatin Organization in Arabidopsis thaliana
Source: G3 (Bethesda). 2017 Jun 6;7(8):2519–31. doi: 10.1534/g3.117.043539 (PMC5555459; doi:10.1534/g3.117.043539)
Supplement: Supplementary file 2 [file 2519FileS2.docx]

**Supplemental Figures and Tables**


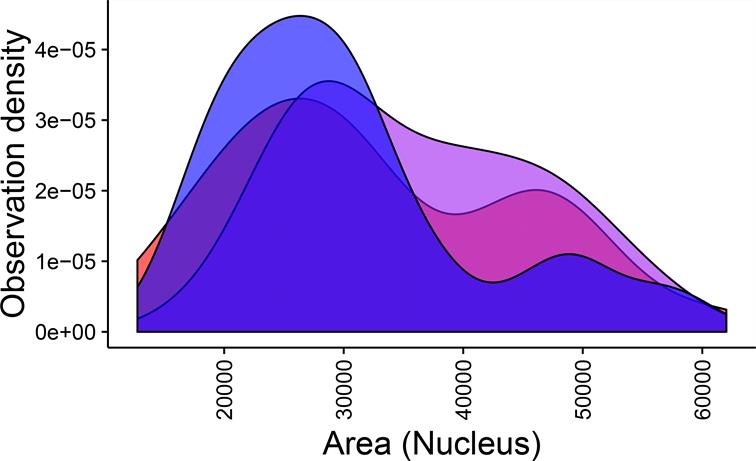


**Figure S1.** **Frequency distribution of observed nucleus *Area* (pixels) values** in a population of individual Cvi-0 (red), L*er* (blue), and F1 hybrid (purple) nuclei.

*
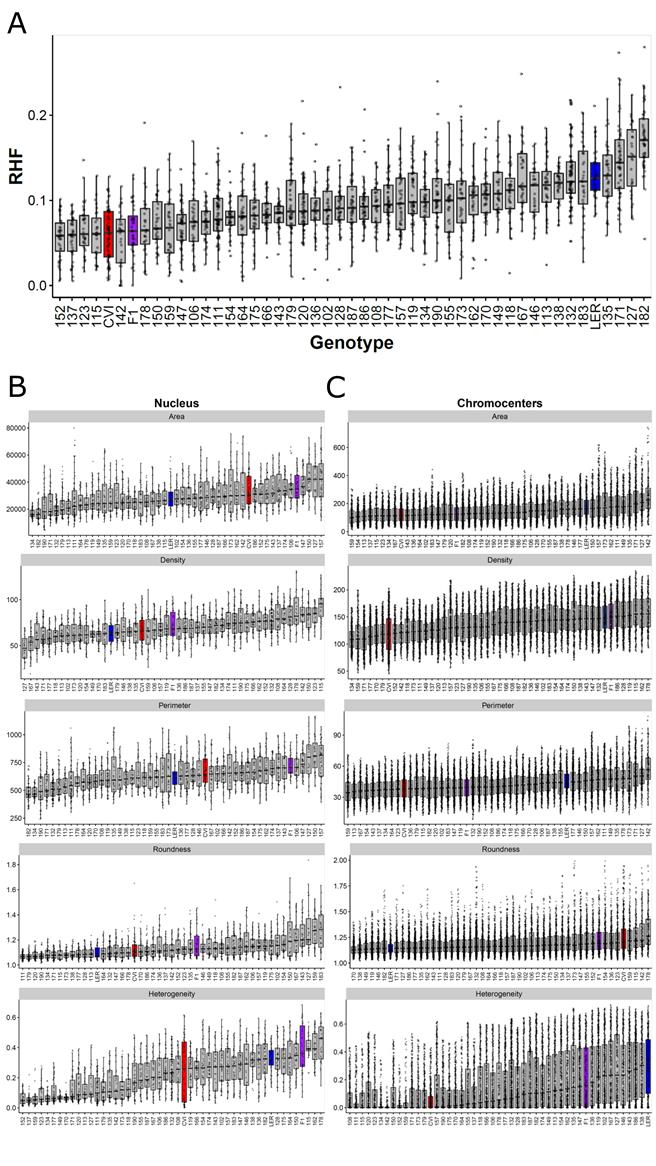
*

**Figure S2.** **Trait effect values of nucleus and chromocenter morphometric parameters** sorted on their trait values, of 46 L*er* x Cvi-0 RILs, parental lines L*er* and Cvi-0 and F1 hybrid. (A) composite *RHF* is from (Tessadori *et al.* 2009). (B) nucleus morphometric parameters, (C) chromocenter morphometric parameters. Outliers beyond two times the SD from the mean per line per trait and tested plant line, were removed prior to sorting on effect. The parental lines are indicated in red (Cvi-0) and blue (L*er*) and hybrid F1 in purple. Black horizontal bars indicate the median. Boxes indicate the boundaries of the second and third quartile and the error bars (whiskers) indicate the values in Q1 and Q4 within 1.5 times the inter-quartile range. Note that substantial transgression exists for most morphometric traits.

**
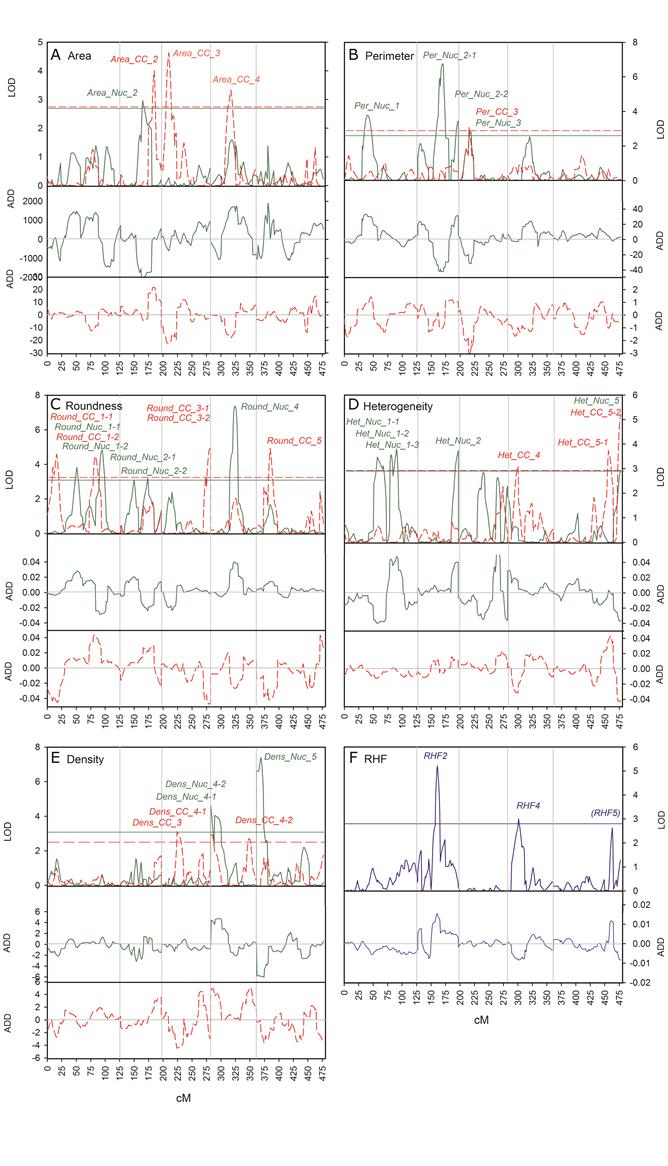
**

**Figure S3. QTL-LOD and additive (ADD) effect profiles** along the chromosomes (cM) of nucleus (green lines) and chromocenters (red lines) morphometric parameters of (A) *Area,* (B) *Perimeter*, (C) *Roundness*, (D), *Heterogeneity* and (E) *Density*. Panel (F) shows the LOD profile and additive effects of *RHF* (in blue; Tessadori *et al.,* 2009) for comparison. In each panel the upper window represents the LOD score profile. The 1000 permutation-determined 95% confidence thresholds are indicated by horizontal green lines (nucleus parameters) and dashed red lines (chromocenter parameters). Middle and lower rows indicate the additive effect of the L*er* allele compared to the population average of respectively nucleus parameters (green; middle row) and chromocenters (red, lower row). QTL names are shown near each QTL. Vertical gray lines indicate chromosomes (chromosome 1-5 from left to right respectively).

**
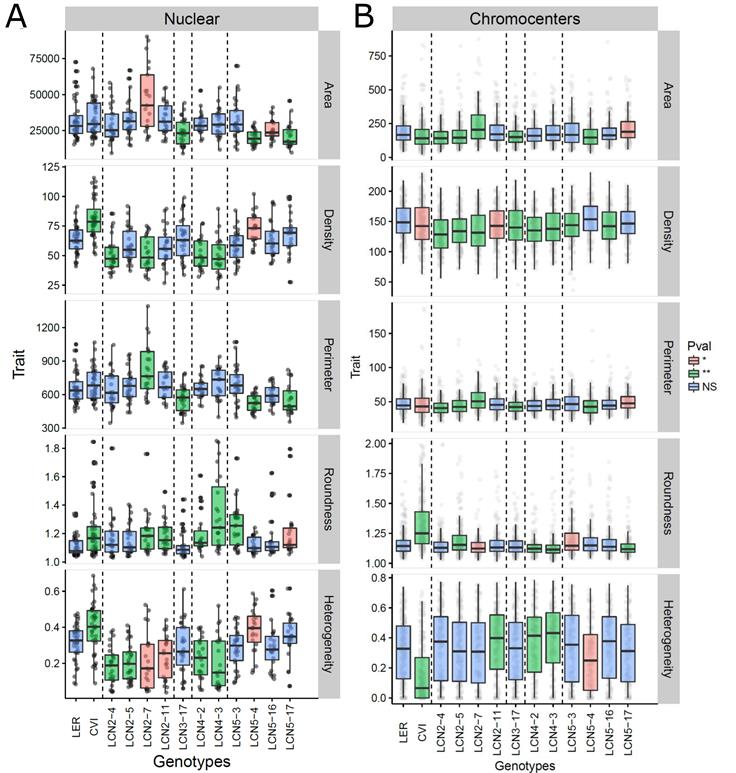
**

**Figure S4. Near Isogenic Lines and parental lines trait effect values** of (A) nucleus and (B) chromocenter morphometric parameters. Black horizontal bars indicate the median. Boxes indicate the boundaries of the second and third quartile and the error bars (whiskers) indicate the values in Q1 and Q4 within 1.5 times the inter-quartile range. Observations outside 1.5 times the interquartile range are indicated as dots. Significance values are reflected in the bar colors. Blue bars = non-significantly different from L*er,* orange bars *p*=0.05 (*), green bars = *p*=<0.01 (**). Vertical dashed lines separate the NILs on chromosome of the main Cvi-0 introgression.


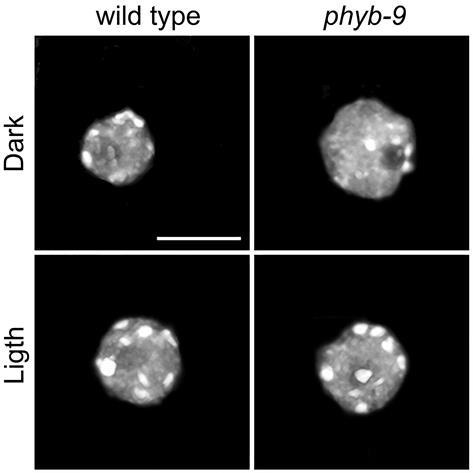


**Figure S5. Etiolated cotyledons of *phyb-9* mutants exhibit large nuclei.** (A) Representative picture of DAPI-stained nuclei of wild-type Col-0 and *phyb-9* cotyledon nuclei obtained from 5-day-old etiolated seedlings constantly grown in darkness (Dark) or shifted to white light (100 µmol m^-2^ s^-1^) for the last 24 hours (Light). Bars correspond to 5 µm.

**Table S1. Estimation of normal distribution of chromocenter and nucleus morphometric traits.**

| **Trait** | **Nucleus** | **Chromocenters** |
| --- | --- | --- |
| *Area* | ns (*p* = 0.64) | ** (*p* = 0.0022) |
| *Density* | ns (*p* = 0.74) | ns (*p* = 0.029) |
| *Perimeter* | ns (*p* = 0.65) | ** (*p* = 0.0066) |
| *Roundness* | ** (*p* = 0.0013) | *** (*p* = 0.000031) |
| *Heterogeneity* | ns (*p* = 0.062) | * (*p* = 0.010) |

***Footnote:*** Values represent *p*-values of significance determined by a *Shapiro* test or normality. Significant *p*-values indicate significant difference from a normal distribution. ns = not significant, * *p*<0.05, ** *p*<0.01, *** *p*<0.001.

**Table S2. Significance (*p*) values of the Pearson correlations between averaged trait values obtained from the RILs, parental lines and F1 hybrid.**

| **Nucleus** |  |  |  |  |  |
| --- | --- | --- | --- | --- | --- |
|  | **RHF** | Area | Density | Perimeter | Roundness |
| Area | 0.004646636 |  |  |  |  |
| Density | 0.000116805 | 0.76483921 |  |  |  |
| Perimeter | 0.00809732 | 1.81E-31 | 0.538054958 |  |  |
| Roundness | 0.522450742 | 0.005510685 | 0.012501045 | 2.91E-05 |  |
| Heterogeneity | 0.74692641 | 0.197208827 | 0.006559751 | 0.12692422 | 0.475247742 |
|  |  |  |  |  |  |
| **Chromocenters** | |  |  |  |  |
|  | **RHF** | Area | Density | Perimeter | Roundness |
| Area | 0.043518859 |  |  |  |  |
| Density | 0.992281964 | 0.127490269 |  |  |  |
| Perimeter | 0.21028073 | 9.07E-38 | 0.165245592 |  |  |
| Roundness | 1.59E-05 | 0.655997333 | 0.87226616 | 0.667905777 |  |
| Heterogeneity | 0.0009167 | 0.788189023 | 6.70E-06 | 0.54371161 | 0.016457658 |
|  |  |  |  |  |  |
|  |  |  |  |  |  |
|  | **Chromocenters** |  |  |  |  |
| **Nucleus** | Area | Density | Perimeter | Roundness | Heterogeneity |
| Area | 0.055415957 | 0.263482773 | 0.041139837 | 0.3493492 | 0.373044903 |
| Density | 0.54472094 | 0.010868668 | 0.988533964 | 0.002197865 | 0.001152942 |
| Perimeter | 0.217992719 | 0.111674577 | 0.180419833 | 0.343230024 | 0.138963196 |
| Roundness | 0.64408751 | 0.259306912 | 0.498433613 | 0.92651335 | 0.022718629 |
| Heterogeneity | 0.426105659 | 6.81E-16 | 0.556604705 | 0.232459512 | 0.003435813 |

***Footnote:*** *p* values of the differences in morphometric parameters of nucleus (upper table), chromocenter (middle table) and nucleus vs. chromocenter (lower table), as presented in Figure 4 and Table 2, are shown. For chromocenter traits, the data is based on the averaged values per nucleus, before the average per line was calculated. Significant *p* values are shown in different shades of red.

**Table S3. Heritability estimates (H^2^) of nucleus and chromocenter morphometric traits.**

|  | **Individual chromocenter** | **Chromocenter Averaged per nucleus** | **Nucleus** |
| --- | --- | --- | --- |
| Area | 8.34 | 25.88 | 14.05 |
| Perimeter | 7.56 | 24.76 | 17.64 |
| Roundness | 1.80 | 10.80 | 21.49 |
| Heterogeneity | 8.32 | 27.25 | 38.54 |
| Density mean | 4.85 | 18.98 | 26.26 |
| ***RHF*** |  |  | 40 |

***Footnote:*** Heritability (%) was estimated by the proportion of between line trait variance divided by the total trait variance. Variance was calculated by a general linear model by means of an *ANOVA* type III.

**Table S4.** **LOD thresholds values** **used for QTL detection at *p<*0.05, determined by 1000 permutation test.**

|  | **Chromocenters** | **Nucleus** |
| --- | --- | --- |
| Area | 2.78 | 2.76 |
| Density mean | 2.61 | 2.91 |
| Perimeter | 2.92 | 2.65 |
| Roundness | 3.12 | 3.07 |
| Heterogeneity | 2.85 | 2.87 |
| *RHF* |  | 2.89 |

**Table S5. Cvi-0 introgression positions of NILs used to confirm the nucleus and chromocenter morphometric QTLs**.

|  | **1^st^ introgression (cM)** | | | | **2^nd^ introgression (cM)** | | | | | **3^rd^ introgression (cM)** | | | | | | |
| --- | --- | --- | --- | --- | --- | --- | --- | --- | --- | --- | --- | --- | --- | --- | --- | --- |
| **NIL** | **L*er*^a^** | **Cvi-0^b^** | **Cvi-0^c^** | **L*er*^d^** | **L*er*^a^** | **Cvi-0^b^** | **Cvi-0^c^** | | **L*er*^d^** | **L*er*^a^** | | **Cvi-0^b^** | | **Cvi-0^c^** | | **L*er*^d^** |
| **LCN 2-4** | 141.6 | 142 | 166 | 162 |  |  |  |  | |  |  | |  | |  | |
| **LCN 2-5** | 148.2 | 152.6 | 162 | 160.9 | 325.9 | 330.9 | 337.9 | 332 | |  |  | |  | |  | |
| **LCN 2-7** | 156.3 | 160.9 | 197.7 | 190.4 |  |  |  |  | |  |  | |  | |  | |
| **LCN 2-11** | 173.2 | 174.5 | 200.9 | 197.7 |  |  |  |  | |  |  | |  | |  | |
| **LCN 3-17** | 270.1 | 276.2 | 288.5 | 288.5 | 297.5 | 311.4 | 322.5 | 316.3 | | 439.5 | 439.5 | | 444.7 | | 441.4 | |
| **LCN 4-2** | 247 | 247.6 | 253.5 | 250.5 | 270.1 | 276.2 | 288.4 | 288.1 | | 290 | 290 | | 297.5 | | 290 | |
| **LCN 4-3** | 110 | 111.9 | 117.4 | 115.3 | 312.1 | 316.3 | 337.9 | 332 | |  |  | |  | |  | |
| **LCN 5-3** | 392.3 | 395 | 421.9 | 419 |  |  |  |  | |  |  | |  | |  | |
| **LCN 5-4** | 111.9 | 115.3 | 121.1 | 117.4 | 383.6 | 388.4 | 395 | 392.3 | |  |  | |  | |  | |
| **LCN 5-16** | 156.3 | 160.9 | 173.2 | 171 | 456.4 | 459.4 | 480.4 | 479.6 | |  |  | |  | |  | |
| **LCN 5-17** | 181.5 | 184.9 | 197.7 | 189.2 | 463.1 | 464.8 | 483.2 | 482.5 | |  |  | |  | |  | |

***Footnote:*** NILs contain a Cvi-0 introgression in the L*er* genetic background and are derived from (Keurentjes *et al.* 2007). Per introgression (1^st^, 2^nd^ and 3^rd^, if present) the flanking L*er* and Cvi-0 markers are indicated (in CM). ^a^ Closest L*er* marker at the left side of the introgression, ^b^ Outer Cvi-0 marker of the left side of the insert. ^c^ Outer Cvi-0 marker of the right side of the introgression, ^d^ closest L*er* marker at the right side of the introgression.

**Table S6. Significance (*p*) values of trait effects in individual NILs compared to the L*er* parental background**.

|  | **Nucleus** |  |  |  |  |  |  |
| --- | --- | --- | --- | --- | --- | --- | --- |
|  |  | **Area** | **Density** | **Perimeter** | **Roundness** | **Heterogeneity** |  |
|  | **Cvi-0** | 0.5773 | 5.05E-06 | 0.1893 | 0.0035 | 0.0024 |  |
|  | **LCN2-4** | 0.4218 | 0.0007 | 0.7356 | 0.0784 | 1.21E-05 |  |
|  | **LCN2-5** | 0.5821 | 0.1507 | 0.4607 | 0.0986 | 4.60E-05 |  |
|  | **LCN2-7** | 0.0227 | 0.0035 | 0.0069 | 0.0033 | 0.0230 |  |
|  | **LCN2-11** | 0.4877 | 0.1585 | 0.2254 | 0.0040 | 0.0145 |  |
|  | **LCN3-17** | 0.0058 | 0.5840 | 0.0087 | 0.8539 | 0.3827 |  |
|  | **LCN4-2** | 0.9854 | 0.0011 | 0.5414 | 0.0039 | 0.0059 |  |
|  | **LCN4-3** | 0.8705 | 0.0009 | 0.1898 | 2.84E-05 | 0.0038 |  |
|  | **LCN5-3** | 0.9428 | 0.0884 | 0.2667 | 1.19E-05 | 0.2489 |  |
|  | **LCN5-4** | 2.25E-06 | 0.0121 | 1.13E-05 | 0.5269 | 0.0201 |  |
|  | **LCN5-16** | 0.0277 | 0.8261 | 0.1001 | 0.2097 | 0.4769 |  |
|  | **LCN5-17** | 0.0002 | 0.1838 | 0.0079 | 0.0153 | 0.2371 |  |
|  |  |  |  |  |  |  |  |
|  | **Chromocenter** |  |  |  |  |  |  |
|  |  | **Area** | **Density** | **Perimeter** | **Roundness** | **Heterogeneity** |  |
|  | **Cvi-0** | 3.94E-05 | 0.0120 | 0.0331 | 1.13E-33 | 3.24E-20 |  |
|  | **LCN2-4** | 1.99E-05 | 7.24E-13 | 2.06E-06 | 0.0637 | 0.1306 |  |
|  | **LCN2-5** | 0.0008 | 2.93E-09 | 0.0034 | 0.0049 | 0.8518 |  |
|  | **LCN2-7** | 8.61E-05 | 2.28E-07 | 0.0007 | 0.0161 | 0.8768 |  |
|  | **LCN2-11** | 0.7444 | 0.0158 | 0.9570 | 0.3106 | 0.0046 |  |
|  | **LCN3-17** | 0.0001 | 0.0009 | 0.0002 | 0.2150 | 0.5915 |  |
|  | **LCN4-2** | 0.1106 | 1.90E-06 | 0.0807 | 0.0004 | 0.0069 |  |
|  | **LCN4-3** | 0.9346 | 0.0004 | 0.6588 | 2.36E-05 | 0.0002 |  |
|  | **LCN5-3** | 0.8542 | 0.0059 | 0.9236 | 0.0408 | 0.1585 |  |
|  | **LCN5-4** | 0.0028 | 0.2773 | 0.0049 | 0.0778 | 0.0115 |  |
|  | **LCN5-16** | 0.5257 | 0.0038 | 0.7175 | 0.8370 | 0.0591 |  |
|  | **LCN5-17** | 0.0174 | 0.2215 | 0.0318 | 0.0009 | 0.7763 |  |

***Footnote:*** Significance (*p*) values for each measured nucleus and chromocenter morphometric trait as presented in Table 4 and Figure S4.
